# Glial Cell-Based Vascular Mechanisms and Transplantation Therapies in Brain Vessel and Neurodegenerative Diseases

**DOI:** 10.3389/fncel.2021.627682

**Published:** 2021-03-26

**Authors:** Yingying Zhao, Shuanglin Wang, Xiaopeng Song, Junliang Yuan, Dong Qi, Xiaohuan Gu, Michael Yaoyao Yin, Zhou Han, Yanbing Zhu, Zhandong Liu, Yongbo Zhang, Ling Wei, Zheng Zachory Wei

**Affiliations:** ^1^Beijing Clinical Research Institute, Beijing, China; ^2^Department of Anesthesiology, Emory University School of Medicine, Atlanta, GA, United States; ^3^Department of Critical Care Medicine, Airport Hospital of Tianjin Medical University General Hospital, Tianjin, China; ^4^Department of Cardiovascular Thoracic Surgery, Tianjin Medical University General Hospital, Tianjin, China; ^5^Institute of Neurology, Tianjin Medical University General Hospital, Tianjin, China; ^6^Mclean Imaging Center, Harvard Medical School, McLean Hospital, Belmont, MA, United States; ^7^Department of Neurology, Institute of Mental Health, Peking University Sixth Hospital, Beijing, China; ^8^Division of Cardiovascular Medicine, Department of Internal Medicine, University of Utah School of Medicine, Salt Lake City, UT, United States; ^9^Division of Cardiology, Emory University School of Medicine, Atlanta, GA, United States; ^10^Department of Chemistry, The Scripps Research Institute, La Jolla, CA, United States; ^11^Emory Specialized Center of Sex Differences, Emory University, Atlanta, GA, United States

**Keywords:** neurovascular unit, blood brain barrier, vascular neurology, ischemic stroke, local cerebral blood flow, vascular progenitor cells, astrocyte activation

## Abstract

Neurodevelopmental and neurodegenerative diseases (NDDs) with severe neurological/psychiatric symptoms, such as cerebrovascular pathology in AD, CAA, and chronic stroke, have brought greater attention with their incidence and prevalence having markedly increased over the past few years. Causes of the significant neuropathologies, especially those observed in neurological diseases in the CNS, are commonly believed to involve multiple factors such as an age, a total environment, genetics, and an immunity contributing to their progression, neuronal, and vascular injuries. We primarily focused on the studies of glial involvement/dysfunction in part with the blood-brain barrier (BBB) and the neurovascular unit (NVU) changes, and the vascular mechanisms, which have been both suggested as critical roles in chronic stroke and many other NDDs. It has been noted that glial cells including astrocytes (which outnumber other cell types in the CNS) essentially contribute more to the BBB integrity, extracellular homeostasis, neurotransmitter release, regulation of neurogenic niches in response to neuroinflammatory stimulus, and synaptic plasticity. In a recent study for NDDs utilizing cellular and molecular biology and genetic and pharmacological tools, the role of reactive astrocytes (RACs) and gliosis was demonstrated, able to trigger pathophysiological/psychopathological detrimental changes during the disease progression. We speculate, in particular, the BBB, the NVU, and changes of the astrocytes (potentially different populations from the RACs) not only interfere with neuronal development and synaptogenesis, but also generate oxidative damages, contribute to beta-amyloid clearances and disrupted vasculature, as well as lead to neuroinflammatory disorders. During the past several decades, stem cell therapy has been investigated with a research focus to target related neuro-/vascular pathologies (cell replacement and repair) and neurological/psychiatric symptoms (paracrine protection and homeostasis). Evidence shows that transplantation of neurogenic or vasculogenic cells could be achieved to pursue differentiation and maturation within the diseased brains as expected. It would be hoped that, via regulating functions of astrocytes, astrocytic involvement, and modulation of the BBB, the NVU and astrocytes should be among major targets for therapeutics against NDDs pathogenesis by drug and cell-based therapies. The non-invasive strategies in combination with stem cell transplantation such as the well-tested intranasal deliveries for drug and stem cells by our and many other groups show great translational potentials in NDDs. Neuroimaging and clinically relevant analyzing tools need to be evaluated in various NDDs brains.

## Introduction

Sudden onset of neurodevelopmental and neurodegenerative diseases (NDDs) with severe neurological/psychiatric symptoms is primarily caused by multiple factors (acute neuronal injury, age, environment, genetics/sex, and immunity) in CNS and brain vascular disorders (Aschner and Costa, 2015). Astrocytes, the most abundant populations in the brain calculated by cell number, have been either the primary cause of NDDs or a critical player after the onset of CNS disorders (Verkhratsky and Parpura, 2016). Importantly, astrocytes are part of the cellular components of neuronal and neurovascular connections within the brain.

**FIGURE 1:**
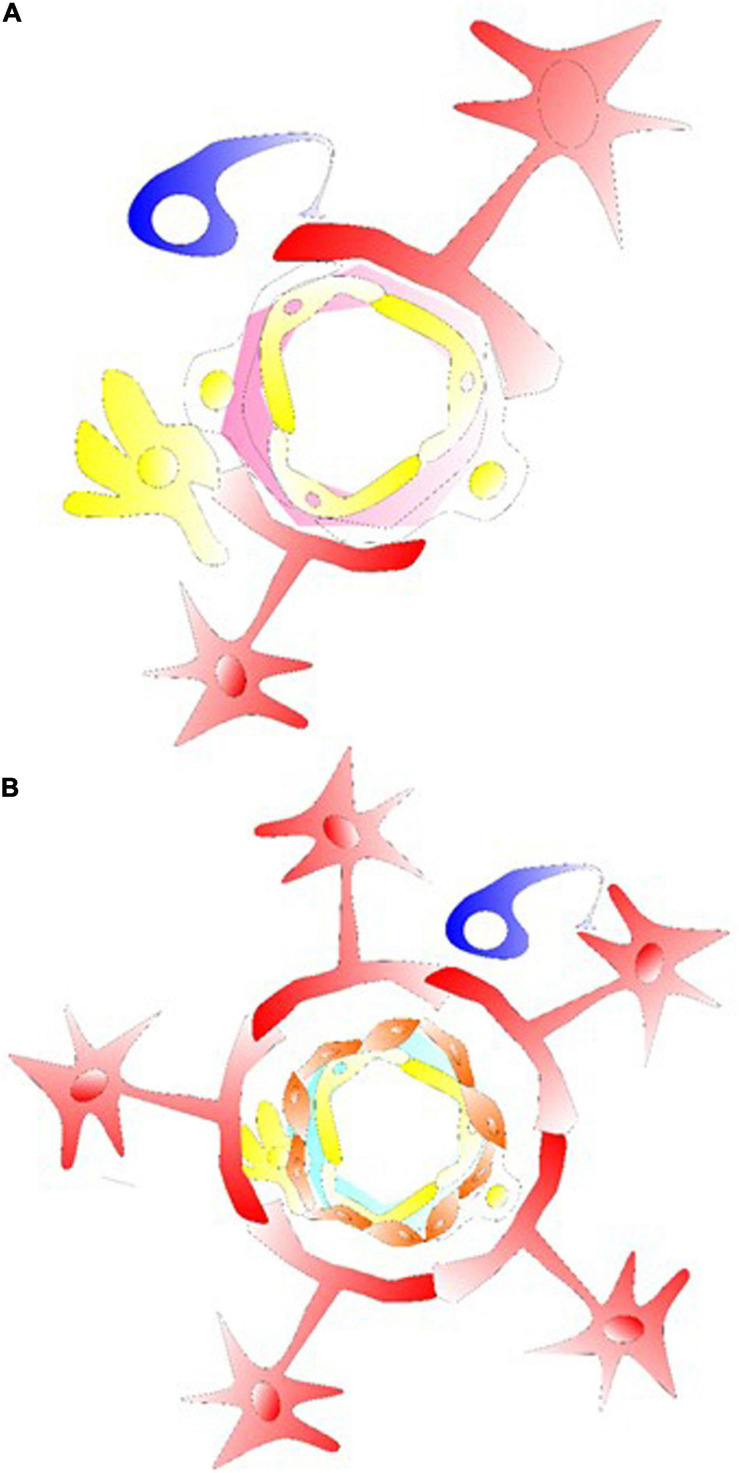
Glial cells in their gliovascular unit. **(A)** The star shaped astrocyte (labeled red) with its end feet on the NVU and capillary component. Together with pericytes, astrocytes usually demonstrate the ability to regulate the capillary tone. This type of astrocyte can interact with pericyte or microglia/macrophage at the capillary levels, inducing an intracellular Ca^2+^ increase via astrocytic P2X. Structures of tripartite synapses are not shown here, consisting of connecting presynaptic and postsynaptic compartments and their enveloping astrocytic process that may facilitate the mechanisms of neurotransmission and astrocytic Gaq/Ga11 GPCR signaling for Ca^2+^-dependent release of gliotransmitters and synaptic modulation via diacylglycerol kinase and PLD2-AA. **(B)** For astrocyte-arteriolar molecular crosstalk, the BBB, or at levels of neurovascular coupling, an endothelium vascular active mediator such as NO releases can affect the VSMC layer (labeled orange) such as to cause VSMC relaxation and arteriolar dilation. RBC is not labeled in the graph, but together with endothelial cells that under shear stress release ATP in the vessel to interact with endothelium P2X/P2Y generating/activating AA, cAMP/PGI2, eNOS, PGE2 and EETs. In here, the endothelial receptor may be targeted with acetylcholine, adenosine, ADPs, ATPs, bradykinin, and UTPs activating cAMP/PLA2/PLC, diacylglycerol-AA/EETs/PGI2 to cause VSMC relaxation. Astrocytic/neuronal ATPs are released activating VSMC P2X, P2Y, intracellular Ca^2+^ to induce VSMC contraction for vasoconstriction.

Moreover, at the end of the smallest arteries and intracerebral parenchymal arterioles, there may be wrapped astrocytic processes and end feet walls instead of cellular structures being in direct contact with the perivascular nerves within the vascular component structure called the basolateral surface.

(Huang et al., 2019) Research accumulated during these decades have supported, anatomically, and functionally, the concept of neurovascular coupling; and more recently that of a gliovascular unit. In those structural units, Ca^2+^ signals broadly activate pathways leading to dilation of the pial arteries/penetrating arterioles (Straub and Nelson, 2007). It also involves releasing various factors into local environment containing adenosine/ATPs, K^+^, prostaglandins, EETs, or some others, differentially regulated from neuronal releases of NO, prostanoids, and vasoactive neuromodulators/neurotransmitters. Astrocytes (such as in their cellular processes) contain one of the main active sources of Ca^2+^ in the brain. Therefore, it has been hypothesized that astrocytes, endothelial cells, myocytes, and neurons form units of neuro-/gliovascular integrations and the adjacent capillary component, which may collectively affect the local cerebral blood flow (LCBF) (Figure 1 and Supplementary Figure S1). During neurodegeneration, there have been reduced LCBF and local hypoxia/ischemia-like changes as degenerative conditions. The review focuses on the cerebrovascular changes/events of some NDDs (e.g., AD, CAA, chronic stroke) for the understanding of vascular mechanisms as well as proposed mechanisms of cell-based therapies (including but not limited to glial cell treatment).

Mitochondria in the cells are actively maintained for continuous actions of an oxidation with energy substances, which are critically supplied from the blood flow, ultimately for cellular and physiological functions (Wei and Wei, 2017). Since neural cells and neuronal activities use ATP hydrolysis and normally consume that energy to maintain the ionic gradients of crossing plasma membranes, we believe that in acute and chronic energy deficient conditions, there are shared cellular physiological and pathobiological mechanisms behind NDDs and brain involvement. During ischemia, there is always rapid impairment of an oxygen supply, an ATP production, and exhaust energy substances. This ischemic condition will likely block mitochondrial respiration and disrupt glycolysis-related activity, which are generally two major energy metabolism processes in the cells. Restored LCBF shortly after an ischemic stroke can immediately initiate energy production of ATPs and uses of other energy substances/metabolites. As the recovery of an energy demand within the brain tissues after ischemia, it is provided regardless of probable regional differences in the sensitivity for an ischemic insult, with delayed restoration of the ATP content for hours (Rosenkranz et al., 1986). Following ischemic reperfusion, alterations in mitochondrial dynamics have been related to an acute cell death, chronic hypoxia/ischemia, or leading to cerebrovascular changes/events for NDDs. Ca^2+^ homeostasis inside mitochondria may be disrupted, followed by impaired mitochondrial respiration and ATP production. This links to the primary cause of energy deficiency in cells of post ischemic brains (Miller et al., 1980). Regulation of phosphate related receptors such as mitochondrial permeability transition pore (mPTP), NCX, S1PR, and VDACs (Supplementary Table S1 for full term), which are usually precisely controlled, can play significant dual roles in reperfusion injury (Morciano et al., 2017). Ca^2+^ is also involved in maintaining an electrical potential for an inner mitochondrial membrane. In hypoxic/ischemic conditions both acutely and chronically, an inefficient Na^+^/K^+^ ATPase activity will be associated with cytoplasmic Na^+^ accumulation and NCX-mediated Ca^2+^ influx and intracellular Ca^2+^ increases. The intracellular and the ER/SR Ca^2+^, critically regulated in cellular processes, can also affect the Ca^2+^ dynamics in mitochondrial compartments. At the same time, during the restoration of mitochondrial respiratory activity at an early ischemic reperfusion, there are continuous increases in intracellular Ca^2+^ concentration. An increase in mitochondrial Ca^2+^ is generally observed, and the levels can be reversed at the first hour of ischemic reperfusion compared with it during ischemia; there are some ischemia-susceptible region-specific delays in mitochondrial Ca^2+^ increase for as long as 24 h later in the reperfusion (Kristián and Siesjö, 1998). The whole event can thereby cause significant ATP loss and lead to energy deficiency, which has been considered as one of the key mechanisms in the progressive pathobiology behind an ischemic reperfusion injury of the brain, where the ER/SR-mitochondrial mechanisms as well as Ca^2+^ movement involvement are critical (Kristián and Siesjö, 1998).

In types of smooth muscle cells including VSMC, IP3Rs, and RyRs families of SR Ca^2+^ release channels identified to date take primary roles in the SR Ca^2+^ release and for contraction/relaxation, differentiation, and proliferative responses. Inside the neurovascular unit (NVU) and capillary component, roles of the ER/SR have been well clarified, dependent on molecular mechanisms of IP3Rs and RyRs to regulate parenchymal arterioles as well as SR regulation of intracellular Ca^2+^. Constantly, SR is involved in the release and re-uptake of Ca^2+^, and maintenance of Ca^2+^ homeostasis in smooth muscles. Normally in VSMC, intracellular Ca^2+^ concentration is estimated to be around 120 nM at the lower 10 mmHg intraluminal pressure. SERCA is primarily responsible for transporting Ca^2+^ back to the SR stores, whereas calreticulin, calsequestrin, and some other Ca^2+^ binding proteins within the SR lumen facilitate an active Ca^2+^ pumping against its gradient and reduce luminal Ca^2+^ (Song et al., 2007). SR Ca^2+^ can be released into cytosol through the opening of SR Ca^2+^ channels. Vasodilation depends on hyperpolarized VSMC membrane to deactivate VOCCs and decrease intracellular Ca^2+^. The level of VSMC cytoplasmic intracellular Ca^2+^ should basically control the parenchymal arteriolar diameter and maintain VSMC/vascular tone. In the flow control, since an endothelial-mediated arteriole-regulating mechanism has been well evidenced elsewhere, it needs to be confirmed considering those mechanisms in the regulation of brain functional arteriole vasculature. Neurovascular coupling, contractility of VSMC, and various arteriolar dilation mechanisms (both an endothelial-dependent and an endothelial-independent) can become dysfunctional when demonstrated in NDDs, such as in models of AD and PD with early symptoms of mild cognitive impairment (MCI), CAA, chronic stroke including post stroke depression, and vascular dementia. Although there is evidence, it needs to be confirmed how energy and molecules involved in astrocytes and glial limitans are important in chronic progression of the diseases. We attempt to include the recent discussion on these astrocytes that may be different from the reactive astrocytes (RACs).

Since NDDs have attracted greater attention, with the incidence and prevalence markedly increased over the past few years, the brain involvement, where it appears as significant neuropathologies especially being observed in neurological diseases in the CNS, are commonly believed to be caused by multiple factors such as an age, genetics/sex variables, an immunity, and a total environment contributing to their progression and neuronal injury. In our research, our group primarily focused on the studies of glial involvement/dysfunction in part with the blood-brain barrier (BBB) and the NVU changes, which have been suggested as critical roles in many NDDs. It has been noted that glial cells, including astrocytes (outnumbering other cell types in the CNS), essentially contribute more to the BBB integrity, extracellular homeostasis, neurotransmitter release, regulation of neurogenic niches, and synaptic plasticity in response to neuroinflammatory stimulus and injury. Behind these, LCBF and the BBB functions are critically important to deliver oxygen and glucose to where it is needed. Considering those, OGD has been a well-accepted model and can provide mechanisms of neuronal injury mimicking hypoxic/ischemic conditions of acute and chronically progressed changes as well as aging-induced mechanisms (Wang et al., 2018). *In vivo* LCBF, primarily supported from small arteries, arterioles, and some capillaries, is maintained in a more uniform manner and coordinately controlled among interconnected blood vessels through the actions of molecular signals and neuronal activity. More specifically, neurovascular coupling is one of the mechanisms by which LCBF is dynamically regulated to match with local brain activity needs. A recent study of NDDs utilizing brain imaging, cellular and molecular probing, and genetic and pharmacological tools has demonstrated the role of RACs and gliosis, the ability to trigger pathophysiological/psychopathological detrimental changes to the disease progression (Michinaga and Koyama, 2019). In particular, the BBB, the NVU and capillary component, and RACs not only interfere with neuronal development and synaptogenesis, but also generate oxidative damages, contribute to Aβ deposition/clearance balance, as well as cause neuroinflammatory disorders (Li et al., 2011). The current review will not primarily focus on the significant roles of RACs (neuroinflammatory and neurotoxic effects), instead, due to the migratory/morphological/functional switches of astrocytes to become RACs, vessels from the BBB, the NVU, and astrocyte-containing vasculatures are expected to be modulated with chronic changes affecting the brain vasculature (in our view). Consistently, the interacted pericytes and VSMC will be also included and discussed here.

**FIGURE 2:**
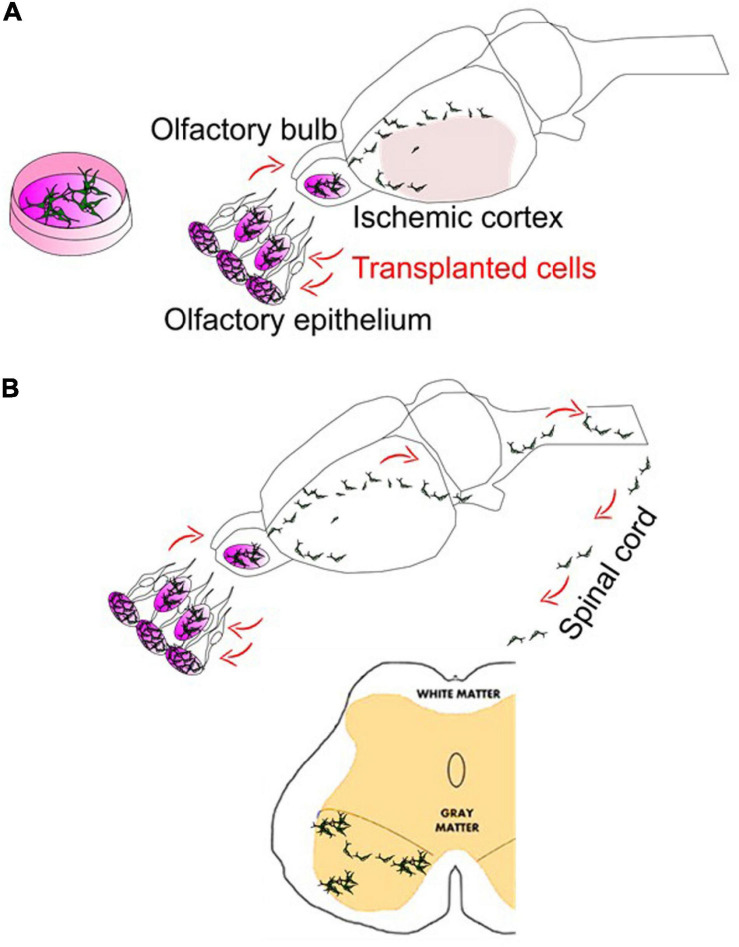
Intranasal delivery to bypass the BBB in stroke and ALS. **(A)** Intranasal delivery of stem cells following cerebral ischemia can show the robust deliveries into the mouse/rat brains. In addition, although not shown here, stem cells intranasally delivered may hold the potentials and repair the vasculature of the brain (Sun et al., 2015). **(B)** The cells intranasally delivered have demonstrated the ability to reach the spinal cord. Meanwhile, there are many trials on the use of spinally targeted cellular treatment for amyotrophic lateral sclerosis (ALS) or spinal cord injury (SCI), which have reached phase I and phase II clinical stages. Intranasal delivery of drugs such as growth factors or stem cell-derived exosomes have been recently tested in ALS mice models (Zhong et al., 2017; Bonafede et al., 2020).

During the past several decades, cell-based therapy has been investigated with a research focus to target related neuro-/vascular pathologies (cell replacement and repair) and neurological/psychiatric symptoms (paracrine protection and homeostasis). Evidence has shown that transplantation of neurogenic or vasculogenic cells could be achieved to pursue differentiation and maturation within the diseased brains as originally expected following transplantation. It would be hoped that, via regulating functions of astrocytes, astrocytic involvement, modulation of the BBB, the NVU and astrocytes should be one of the major targets for therapeutics against NDDs-like pathobiology by drug and cell-based therapies. Preconditioning strategies (Yu et al., 2012; Wei L. et al., 2017; Wei Z. Z. et al., 2017; Wei et al., 2019) tested by our and many other groups in combination with stem cell transplantation and the well-tested intranasal deliveries for drug and stem cells (Wei et al., 2016; Shen et al., 2020) show great translational potential in NDDs (Figure 2). Altogether, the glial cell-based therapy might have included principles of: (1) targeting molecular/morphological changes from glial cell populations at particular time courses/diseased progression periods (Zhang et al., 2018); (2) transplanting glial cell-differentiated/adopted stem/progenitor cells; (3) *in vivo* reprogramming of glial cell subtypes (Masserdotti et al., 2015). We and others attempt to investigate the potential benefits of those glial cell-based therapeutics. As in our and others’ research, it focuses on a quite promising topic that can be broadly adapted in neurologic diseases considering with few major advances in other therapeutic approaches, glial cell-based therapy and cell transplantation should be a new attempt for these diseases.

### Blood Flow, Ca^2+^ Movement, Glial Cell, and RyRs

Innovative brain imaging, and the disease mechanisms revealed by this, have been introduced in recent years. More neuroimaging techniques and clinically relevant analyzing tools will be needed to develop a potential treatment being continuously monitored and evaluated in NDDs brains (Wei et al., 2018b). One example for NDDs is an ischemic stroke at the chronic phase, one of the leading causes of human death and disability worldwide; stroke in aging and female populations is more frequently causing long-term morbidities. Following cerebral ischemia, for example, it has long been believed that an ischemic reperfusion causes many parallel changes involving mitochondrial activity changes and levels of ATP-producing. Neuroprotection against neuronal loss can be significant in the acute/subacute phases of ischemic stroke patients. At an initial phase after the ischemia, reperfusion itself recovers to support energy metabolism in both astrocytes and neurons and form ischemic penumbra, where the energy-related metabolites and mitochondrial function is recovered (Fordsmann et al., 2019). Ca^2+^ movement from cytoplasm as well as the ER/SR into the mitochondria at particular timing, together with dynamic alteration of oxidative glucose metabolism in the mitochondria, greatly reduce the respiratory capacity and cause energy deficiency. The mitochondrial homeostasis is important as a therapeutic target for stroke and many other NDDs, as it is also widely observed for such dysregulation of mitochondrial functions in degenerative processes after ischemic stroke and NDDs. There are molecular mechanisms in the mitochondrial compartment, energy metabolic changes, ion channel regulations, mitophagy, mitochondrial transfer, and biogenesis during and after ischemia. In addition, pursuing recent techniques of optogenetics, chemogenetics, and optochemogenetics, regulation of neuronal excitability has been promising for treating acute and chronically progressive excitotoxicity by means of ion channel controls and ion flow modifications (Cheng et al., 2014; Gomez et al., 2017).

VSMC is a major type of vascular wall cell in both connecting precapillary arterioles and the BBB, which mostly includes the GVU or astrocytic limitans. Ca^2+^ signaling in both astrocytes and VSMC are active and utilized for their physiological functions. Here we mainly introduce the subcellular mechanisms under IR3R and RyR. The IP3R consists of four subunits with an approximate 310 kDa molecular weight of each. There are three subunit genes of the receptor subtypes that encode the one subunit and form four to become one unit of IP3R. Among the three isoforms, IP3R1 subunit is predominant in adult VSMC, while IP3R2/IP3R3 subunits have been more selectively involved in proliferating VSMC. This type of combination forming IP3Rs heterotetrameric complexes can be as shown in co-immunoprecipitation experiments. Following the Gaq and Ga11 signaling and PLC activation, all IP3/IP3Rs activation have been linked with the cytosolic signals to the ER. IP3Rs can respond to signals of some vasoactive molecules including acetylcholine, endothelin 1, 5-HT, and noradrenaline (Bhaskaran et al., 2014). Interestingly, the IP3Rs-mediated Ca^2+^ release is potentiated at a low concentration of 500 nM Ca^2+^ or below; a higher Ca^2+^ seems to inhibit the Ca^2+^ release but it may be affected by different subtypes. IP3Rs may therefore be involved in the fundamental mechanisms of triggering Ca^2+^ oscillations in astrocytes including RACs when becoming reactive and in VSMC during vasoconstriction. Moreover, in pial arteries and astrocyte end feet, IP3R-mediated Ca^2+^ signaling can activate BK_*Ca*_, TRPC3, or TRPM4 channels (Dunn et al., 2013).

Expressions of RyRs are found in cerebral arteries and in the microcirculation vasculature, where their astrocytes and VSMC are thought to be modulated during micro-environmental and pathologic conditions of NDDs. RyRs are formed by three subtypes of 550–660 kDa subunit each and consist of four subunits to become one unit of RyR. RyR2 may be the main form to have RyR2/RyR3 and RyR2/RyR1 evidenced by an immunoprecipitation and can be located via electron microscopy. RyR1/2 in skeletal muscle or cardiac muscle exert fundamental primary functions for the excitation-contraction coupling. RyR3 can be ubiquitously expressed in many cell types including astrocytes and neurons. Different from IP3R, SR Ca^2+^ load is critical to an open of the channels. Interestingly, RyRs seem primarily regulated by the binding of Ca^2+^ on the cytosolic surface of the channel. Intracellular micromolar Ca^2+^ can induce an opening of RyRs while intracellular millimolar Ca^2+^ shows inhibitions on the opening. SR Ca^2+^ release from neighboring IP3Rs and RyRs can induce the RyRs-mediated Ca^2+^ release as well. All over the body, this Ca^2+^-induced Ca^2+^ release has been thought to be crucial in excitation-contraction coupling of cardiac contraction for cardiac muscle, as well as demonstrated importance for cellular signaling process in non-muscle cell types (e.g., astrocyte or insulin-secreting pancreatic beta cell). In the cerebral arteries including both pial arteries and parenchymal arterioles of the brain, Ca^2+^-induced Ca^2+^ release mechanisms are demonstrated in the types of smooth muscles. Caffeine can activate RyRs and a subsequent transient increase of intracellular Ca^2+^ leading to vasoconstriction. In astrocytes, caffeine treatment may reduce proinflammatory communications with the ER depletion through persistent activations of RyRs. Not likely for cardiac and skeletal myocytes, RyRs-mediated Ca^2+^ release in VSMC during the excitation-contraction coupling limits their global events such that RyRs mediate NVU functionality at levels of parenchymal arterioles during the regulatory cellular events of neurovascular coupling. In response to neuronal activation, these end and smallest arteries and arterioles dilate for increased LCBF. Like in cardiac and skeletal muscle, RyRs-mediated local Ca^2+^ sparks can activate BK_*Ca*_ channels which in the brain oppose VSMC contraction via reducing cell membrane depolarization. For pial arteries, blocking RyRs, and BK_*Ca*_ channels may cause robust and non-cumulative constrictions. Altogether, RyRs and BK_*Ca*_ channels are therefore involved in the negative feedback as a cause of vasodilation. Interestingly, VSMC from pial arteries/parenchymal arterioles, shows one of the significant regulatory Ca^2+^ signals responding to astrocytes, with absent responses from BK_*Ca*_ channel blockers. Under basal conditions, RyRs are also differentially involved in capillaries, arterioles, and pial arteries of the brain. Given increased acidity/H^+^ concentration in the blood and tissues, the RyRs-mediated activation of BK_*Ca*_ channels upon Ca^2+^ sparks leads to vasodilation in parenchymal arterioles and VSMC acidification. In brain slices demonstrating parenchymal arterioles, where the VSMC exhibit synchronous Ca^2+^ oscillations but can be rapidly suppressed with the reach of neurovascular coupling, their VSMC Ca^2+^ can be artificially and effectively affected by the neuronal depolarization when applying electric field stimulation. Astrocytic and neuronal vasoactive substances may also activate Ca^2+^ signals in arterioles. On the brain slice of newborn piglets, arteriolar VSMC also respond to glutamate release (potentially inducing astrocytic NO release) for increased Ca^2+^ spark frequency and decreased cytoplasmic Ca^2+^ (Xi et al., 2010). Similarly, astrocytic heme oxygenase metabolism can generate CO as a vasodilator. CO prevents H_2_S-induced vasoconstriction evidenced in brain slices from the mouse neonate (Morikawa et al., 2012). Involving astrocytes, H_2_S may also exert cytoprotection against hepatic encephalopathy related conditions of cytotoxicity toward apoptosis and oxidative stress (Jin et al., 2020). Inside cells, cytochrome P450 epoxygenase-EETs are introduced to mediate neurovascular coupling events. Considering the mechanisms of action in neurovascular coupling, EETs can induce BK_*Ca*_ channel-mediated hyperpolarization (antagonized depolarization) of VSMC and increase Ca^2+^ spark frequency in pial arteries. EETs may also have modulatory effects of RyRs for neurovascular coupling. For a larger sustained/reversible vasodilation, acidosis can facilitate Ca^2+^ spark-driven BK_*Ca*_ currents of parenchymal arterioles (Dabertrand et al., 2012). Ca^2+^ spark-mediated BK_*Ca*_ channels activation may lead to vasodilation in the presence of CO and EETs contributed to VSMC relaxation. Proton (H^+^) has been proposed to play a role during neurovascular coupling while neuronal proton (H^+^) affects local acidification, associated with vasodilation.

In pathobiological conditions of the brain, where acidosis can induce vasodilation, an astrocytic end foot releases H^+^ onto the VSMC layer to promote the dilation. There is evidence supporting, under cortical stimulation or together with mGluRs activation, an alkalinization of the astrocytic cell bodies. In addition, potential blood mechanisms involved pH buffering-related acid-base homeostasis from the lungs and the kidneys. Such that the lung regulates CO_2_ and kidneys maintain acid-base excretion. CO_2_ inhalation, reduced respiratory rate, as well as hypoxia/ischemia-induced lactic acidosis, will likely lead to acidification in the CNS tissue. Of hypoxic/ischemic conditions, the brain in anaerobic glycolysis is changed with an accumulation of lactic acid and tissue acidification is usually and conservedly obtained after an ischemic stroke. One worst condition in moderate hyperventilation for and clinical management of cerebral ischemia patients may counteract the acidification but lead to increased vascular tone and decreased LCBF. Otherwise, in consideration of endogenous mechanisms during focal cerebral ischemia, acidosis and vasodilation of parenchymal arterioles facilitate perfusion and remodeling of the collateral vessels both acutely and chronically. However, this endogenous LCBF regulatory mechanism seems to be quite dependent on normal astrocyte functionality over RACs from NDDs brains.

Since the brain consumes approximately 20% of the oxygen and glucose from all over the body, and that of the cardiac output at same levels by which it maintains the neuronal activity, then controlling the flow, LCBF, and neurovascular coupling processes is critical to the brain functions. Functional imaging has been developed based on this phenomenon known as rapid functional hyperemia within different brain regions and branches of pial arteries and penetrating arterioles. Astrocytes that have been tightly wound around the vessels play a role because of physical contact. Physiological consumptions of oxygen and the related oxygen diffusion gradient across capillaries/glia limitans is measurable through brain imaging techniques, which has contributed to our understanding of functional neural networks in the brain. As in chronic stroke and some other NDDs, there may be disrupted functional connectivity whereas coactivated brain regions of neuronal activation patterns indicating a disconnection or hyperconnection as well as neurovascular uncoupling showing an imbalance/mismatch between LCBF, oxygen, and/or neuronal activity. These types of mismatches can provide clinically relevant information for both acute stroke and chronic hypoxia/ischemia (Table 1). The recovery of the flow, LCBF, and neurovascular coupling processes is one of the critical mechanisms for stroke recovery. Over the brain microcirculation, the end and smallest arteries and arterioles (accounting for an approximate ratio of 40% among all the cerebral vascular resistance) have been considered important regulators of the flow, LCBF, and neurovascular coupling processes. An arterial vascular diameter controlling by cerebrovascular autoregulation is critical for maintaining constant perfusion from a range of systemic pressures. At normal conditions and after cerebrovascular injuries, pressure helps to induce vasoconstriction of arteries and arterioles. Additionally, during neurovascular coupling, neuronal activities involve several mechanisms by which functional hyperemia occurs within the brain regions as well as for peri-infarct regions of the post-ischemic brain. As a compensatory mechanism demonstrated in ischemic stroke patients, the study of collateral flow as we and many others do also tends to provide important insights for the understanding of intrinsic vascular mechanisms and disease relevant targets in both acute and chronic conditions of NDDs (Wei et al., 2018a). Stem cell therapy may hold the ability for a fast recruitment of collateral vessels.

**TABLE 1:** Brain imaging and general pathological information in acute ischemic stroke.

**Brain imaging tool**	**Role in brief**	**Mechanism**	**Diagnosis and effect**
Non-contrast computed tomography (NCCT)	Exclude ICH	Ischemia	Regular use of neuroimaging
Magnetic resonance imaging (MRI)	Exclude cerebral microbleeds, ICH	Collateral flow	MRI-evident mismatch (diffusion-positive FLAIR-negative lesions)
Magnetic resonance angiography (MRA)/Diffusion weighted imaging (DWI)	Selecting candidates for mechanical thrombectomy; Demonstration of LVO stroke	Infarct core and penumbra	
CT perfusion (CTP) imaging			For substantial diagnostic uncertainty
CT angiography (CTA)			Sensitive to LVO stroke
Digital subtraction angiography (DSA)	Provide critical diagnostic information	Neuro/glio-vasculature	For enhanced diagnostic accuracy Burger et al., 2006
Carotid and vertebral/combined duplex	Exclude vessel tortuosity, etc.	Recanalization	Access to intracranial circulation and vasculature
Transcranial doppler (TCD) ultrasonography/Transcranial color-coded duplex (TCCD)			

**Brain image-based diagnostic methods in ischemic stroke patients have been summarized based on the Guidelines for the Early Management of Patients With Acute Ischemic Stroke: 2019 Update to the 2018 Guidelines for the Early Management of Acute Ischemic Stroke/A Guideline for Healthcare Professionals From the American Heart Association/American Stroke Association, which is endorsed by the Society for Academic Emergency Medicine and The Neurocritical Care Society, and by the American Association of Neurological Surgeons and Congress of Neurological Surgeons and reviewed for evidence-based integrity. In the table, we have added our understanding for the related mechanisms.**

Cerebral cortical arterial and arteriolar component and network starting over the brain surface coordinately split off pial arteries. Within the parenchyma, the pial arteries dive down into narrowing and branching arterioles that further connect to capillaries where it is demonstrated with the NVU, and where the astrocyte end feet (glia limitans) and VSMC layers surround the endothelial cells and form the perivascular Virchow Robin spaces between the CSF and the ISF of brain (Duperron et al., 2018). In the rodents, an LCBF can be measured at levels of pial arteries and arterioles over a part of the brain surface, e.g., using a laser doppler to transcranially scan the barrel cortical flow change (McCrary et al., 2020). Among the cell types, VSMC interacting with astrocytes and pericytes within small arteries and arterioles can regulate flow through changing the vessel diameter, coordinately transducing molecular signals into vascular tones. Typically, the local Ca^2+^ release inside the VSMC of the end and smallest arteries, where there are arterioles usually containing one VSMC layer; narrowing and branching arterioles generates Ca^2+^ sparks and open through SR RyRs on the membrane followed by an activation of the Ca^2+^-sensitive BK_*Ca*_ channels which cause a change in the vascular diameter; these RyRs and BK_*Ca*_ channels can also play a role in the VSMC of pial arteries/penetrating arterioles. Normally during the resting state and physiological conditions, the arterioles and capillaries seem to provide a safe margin of oxygen supplies to the brain since the neuronal activities stimulate arteriolar and capillary dilation in a timely manner. But, the total control of arteriolar and arterial relaxation and contraction may be changed in the chronic pathological conditions, such as conditions of astrogliosis, hypoxia or ischemia in NDDs. Together with pericytes, astrocytes have been demonstrated to have the ability to regulate the capillary tone (Mishra et al., 2016). It is believed that astrocytes mediate the NVU and capillary component pericyte at the capillary levels, inducing an intracellular Ca^2+^ increase in astrocytes through purinergic receptor P2X rather than the Ca^2+^-induced Ca^2+^ release mechanisms (with IP3Rs and RyRs). But rather, the structures of the tripartite synapse consisting of connecting presynaptic and postsynaptic compartments and their enveloping astrocytic process facilitate the mechanisms of neurotransmission and astrocytic Gaq/Ga11 GPCR signaling for Ca^2+^ -dependent release of gliotransmitters and synaptic modulation. The involved activation pathways have included diacylglycerol kinase, PLD2-AA, and some others. Under light-induced physiological stimulation, the Ca^2+^ signals in astrocytes contribute to the capillary diameter regulation instead of the arteriole one, indicating a glial cell-based therapeutic mechanism. In pericytes, AA-related prostaglandin PGE2 or 20-HETE causes relaxation or contraction, respectively (but not with EETs). Upon an activation of P2X/P2Y, in presence of ATP and cellular Ca^2+^ increase, pericytes depolarize and contract. Activations of BK_*Ca*_, SK_*Ca*_, or K_*ATP*_ channels result in K^+^ efflux and hyperpolarization of pericytes, decreasing Ca^2+^ entry through VOCCs. Adenosine released from neural cells such as astrocytes, may also trigger VSMC relaxation, and trigger pericyte relaxation via binding to α1/2-ARs and activation of K_*ATP*_ channels to induce hyperpolarization. Blocking NOS activity is also shown to reduce glutamate release-related capillary dilation. Evidence links the NO for pericyte relaxation and capillary dilation. Altogether, the inhibition of AA conversion to 20-HETE can block depolarization and contraction. For response to endothelial cells, it is believed that there are shared mechanisms of endothelial-derived vasoactive mediators for the contraction/relaxation regulation between pericyte and VSMC. There are regulatory neurotransmitter/gliotransmitter and endothelial signaling pathways. Evidence also includes the degenerative changes of pericytes in some diseased conditions as reported in ALS and during aging (Bell et al., 2010). It has been concluded that pericyte loss, indeed, is consistent with the vascular dysregulation of the studies in pericyte-deficient animals. Whereas, in the pericyte-deficient mice there is pericyte loss and brain vascular damages, such as the BBB disruption leading to reduced capillary perfusion and microcirculation in the brain and involved in the chronic hypoxia (Ahmad et al., 2011). Since astrocyte processes and pericytes are structurally interacted, the reactivation in the form of RACs is expected to cause an arterial/arteriolar vasculature damage as well as to functionally delay the NVU/GVU processing, as chronic detrimental cerebrovascular mechanisms (Table 2).

**TABLE 2:** Astrocyte and pericyte biological processes.

**Molecular signaling**	**Function**		**NDD Pathology**	
	**Pericyte**	**BBB/NVU**	**RAC**	**Other**
PDGF/PDGFR	Activation, differentiation, recruitment	Endothelial transcytosis barrier, integrity	Angiogenesis, inflammation	
Laminins/dystrophin, dystroglycan	Homeostasis	Astrocytic end feet polarization, integrity		Aβ accumulation
ApoE/LRP1	Loss	Breakdown		Aβ aggregation, microhemorrhages, microinfarct
CXCL12/CXCR4		Neurotrophic release		Microglia activation, WML

**Some molecular crosstalk between astrocyte and pericyte has been summarized. Other Wnt crosstalk, JAK-STAT, Notch, Shh may also be involved through the interaction with above molecules.**

### The BBB, Ca^2+^ Signaling, Myogenic Tone, and Pericyte

Normally in pressurized parenchymal arterioles, Ca^2+^ waves of VSMC do not activate their BK_*Ca*_ channels. Acidification may affect the appearance of Ca^2+^ sparks and Ca^2+^ waves, associated with profound, rapid, and reversible vasodilation. Astrocytic Ca^2+^ waves can be a remarkable intracellular Ca^2+^ dynamic and contribute to the unique type of functional intercellular communication (such as proinflammatory communication) within astrocyte networks. Ca^2+^ influx into the VSMC and a subsequent cytoplasmic Ca^2+^ concentration increase can lead to vasoconstriction. Like in astrocytes where Ca^2+^/calmodulin-dependent proteins and kinases show primarily activity/morphological regulatory roles (such as neurotransmitter uptake and gliotransmitter release), the vascular mechanisms include an activation of many Ca^2+^/calmodulin-dependent proteins including myosin light-chain kinase to control smooth muscle fibers initiating smooth muscle contraction. Altogether, it may activate vasomotor pathways, RyRs, and other Ca^2+^ signals and Ca^2+^ waves, etc. In the cerebral parenchyma, an integrity of the BBB is critically important for maintaining neuronal activities through constantly adapting regional flow, LCBF, and neurovascular coupling processes. Arteriolar tone is also critical for local perfusion, which is mainly dependent on the Ca^2+^ influx across VSMC cell surface membrane. Astrocytes may contribute to the regulation of parenchymal arteriolar vascular tone in terms of their participation in neurovascular coupling processes. It may also be influenced by vascular endothelial cells which involve Ca^2+^-dependent processes leading to subsequent vasodilation (an endothelial cell-dependent mechanism).

The microvascular myogenic tone of resistance arteries is partially involved in the microvascular autoregulation within the brain, where small arteries/arterioles demonstrated the ability to constrict or relax depending on changes in an intravascular/intraluminal pressure. In NDDs, the astrocytic involvement has been observed related to the Ca^2+^ changes (Dong et al., 2018). Typically, an intraluminal pressure increase can lead to constriction of the small arteries and arterioles while an intravascular pressure decrease can lead to vasodilation. VSMC related mechanisms, responsible for the vascular myogenic response ensuring nearly constant blood flow under moment-to-moment fluctuations in neuronal excitability and arterial flexibility, reside in pathological conditions such as to cause cerebral arterial stiffness. Pial arteries/penetrating arterioles among terminal VSMC-containing vessel structures connecting to the capillary bed have been thought as main physical mechanisms of capillary perfusion pressure maintenance and therefore exert critical actions within physiological range and in a pathological state. In the brain microcirculation, there are normally limited collateral supply blood flow and perfusion within the neocortex, which are under regulation of myogenic tone (for example involving mechanisms and modulators of myogenic vasoconstriction). The myogenic mechanisms during pressure-induced constriction in blood vascular wall are demonstrated as main regulatory responses, which is not directly affected in the event that endothelium disruption may occur. That pressure-induced constriction, however, has evolved an arterial myocyte surface membrane depolarization as well as an activation of VOCCs-mediating Ca^2+^ influx to vasoconstriction. VOCCs-mediating Ca^2+^ are involved in neuropsychiatry with chronic Ca^2+^ burden increases (Chen et al., 2014). The small arteries can depolarize and constrict with low intravascular pressure and contribute to around one third of the tone, which may be highly relevant to a physiological vasodilation reserve. The dilation capacity of those arterioles and local vasodilators contribute to the regulation of the flow, LCBF, and neurovascular coupling processes, or a functional adaptation/hyperemia, or the vascular resistance.

Ca^2+^ signals of parenchymal arteriolar VSMC are in forms of Ca^2+^ waves rather than Ca^2+^ sparks at normal pH and temperature conditions. Ca^2+^ waves can transmit in astrocytes in response to injurious stimulus. Ca^2+^ spark-driven BK_*Ca*_ current is one of the key processes, linked to a greater level of the myogenic tone at observed parenchymal arterioles than that in pial arteries, even when there is pressure induced constriction potential for intracerebral parenchymal arterioles. Ca^2+^ waves in VSMC of pressurized parenchymal arterioles have a propagating duration of several seconds, as smaller distributed localized events of Ca^2+^ movement. RyRs mediate both Ca^2+^ waves and Ca^2+^ sparks in parenchymal arterioles. Those Ca^2+^ signals (Ca^2+^ sparks and Ca^2+^ waves) can be blocked from the parenchymal arteriole under SR Ca^2+^ depletion induced by cyclopiazonic acid or by tetracaine to block RyRs (Kur et al., 2013). IP3R involvement may also be involved. Ca^2+^ sparks and Ca^2+^ waves have also been presented in cremaster muscle feed arteries and arterioles, where there are differential roles of RyRs and IP3Rs due to subtypes and distribution divergence. On the other hand, perivascular nerves at the arterioles can release Gaq/Ga11 GPCR agonists for the facilitation of Ca^2+^ waves via an IP3R-dependent mechanism, which is also demonstrated in the GVU vasculature and gliotransmission systems. In addition, an activation of RTKs signaling within the cells such as astrocytes and VSMC also generates IP3 for Ca^2+^ waves. Parenchymal arterioles without extrinsic innervation mainly depend on higher cytoplasmic intracellular Ca^2+^ to activate RyRs. There are also quite variable mechanics for Ca^2+^ waves being triggered/propagated in smooth muscles. There is a variation with RyRs functions in-between levels of arteries. For parenchymal arteriolar VSMC, there are predominant Ca^2+^ waves. The effects of RyRs and BK_*Ca*_ channel by natural blockers are considered limited for parenchymal arteriolar diameter controls. Like astrocytic Ca^2+^ waves, which are activated through NMDARs, P2X, and/or GPCRs with an initiation of intracellular Ca^2+^ in astrocytes in quick responses to an acute injury, the Ca^2+^ signals spread fast for the physiological needs (Boitier et al., 1999). The role of RyRs-mediated Ca^2+^ waves for cerebral arteries has been shown to be important for maintaining the myogenic tone under low pressure involving the myosin light-chain kinase (human gene names: *MYLK*) and increased phosphorylated myosin light chain. At lower pressures, parenchymal arterioles can be depolarized and constricted to a greater extent than pial arteries. Some localized Ca^2+^ release events have been demonstrated to have the ability to initiate the Ca^2+^ waves. Normally, Ca^2+^ waves do not deliver sufficient Ca^2+^ that are essential for BK_*Ca*_ channels activation, but Ca^2+^ sparks will deliver micromolar Ca^2+^ for BK_*Ca*_ channels activation. Such that Ca^2+^ spark initiates Ca^2+^ waves at blood vascular beds demonstrated in small mesenteric arteries, retinal arterioles, and the hepatic portal vein. Within a cell, local Ca^2+^ increases may activate the neighboring RyRs (with high resting open state probability). Ca^2+^ sparks initiate Ca^2+^ waves involving the Ca^2+^ induced Ca^2+^ release mechanism (Mulligan and MacVicar, 2004; Kuga et al., 2011). Altered RyR expression has been verified in some NDDs models including AD linked to the amyloid hypothesis and the spinal cord of MND. Observations in VSMC of pial arteries have suggested the RyRs resting open state probability and the intracellular Ca^2+^ signals in regulation of the flow. At low resting open state probability, the local RyRs-mediated Ca^2+^ release in forms of Ca^2+^ spark will not activate neighboring RyRs for an induction of Ca^2+^ wave. IP3/IP3R signals increases in parenchymal arterioles, together with RyRs, induces Ca^2+^ waves. The decrease in intracellular proton (H^+^) levels will likely increase RyRs resting open state probability for an initiation of Ca^2+^ waves. A slight pH value increase (from 7.4 to 7.5) will increase the frequency of Ca^2+^ sparks while at a pH of beyond 7.6 it seems that the Ca^2+^ signals will be shifted from stationary Ca^2+^ sparks to Ca^2+^ waves. In NDDs, significant pH changes of the parenchymal compartment, for example increased pH in HD patients measured by MRI, may also occur during chronic hypoxia/ischemia.

Astrocytic regulation for the flow, LCBF, and neurovascular coupling processes are closely related to their interactions with pericytes (Table 3). There may be heterogeneous pericytic cells depending on combined subtypes within distinct vasculatures, their location, and morphological need in the control of capillary blood flow. In that case, some pericytes have been evidenced to regulate capillary diameter and LCBF. Neurotransmitters (for example, noradrenaline, another significant modulator on astrocytes), electrical stimulation, or neuronal circuit activity can lead to Ca^2+^-dependent pericyte contraction. An example includes potential astrocyte-pericyte-mediated neurovascular coupling of capillaries, as well as retina Muller cells-regulating capillary under optogenetic stimulation in the neurons. Pericyte degeneration or loss, with the similar time course showing astrocytic pathology such as convergent loss of astrocyte end-feet in NDDs, may cause neurovascular uncoupling with reduced O_2_/blood supply into the brain regions. In mice with pericyte-deficiency, it has been demonstrated with diminished hemodynamic responses (Kisler et al., 2017b). Interestingly, independent of neuronal input as we and others previously demonstrated using stimulation methods (Song et al., 2017; Yu et al., 2019), some non-neuronal cell-targeted optogenetic experiments might support that NG2-positive cells do not significantly contribute to LCBF regulation with stimulations under control of channel rhodopsin expression and that PDGFR-β-positive cells (including pericyte) contraction, with constriction of capillary lumens and inhibition of RBC flow. There are potential differences in responses following stimulation via optogenetics in previous studies on potentially different pericyte subpopulations. The example is that, in the mouse sensory cortex, there can be pericyte relaxation, and capillary dilation ahead of arterioles, when exposed to hindlimb stimulation at 10s or whisker pad stimulation at 15s, with pericyte relaxation without dilation of vessel if at 2s. Another example has included, in the rat olfactory bulb of the brain, the olfactory glomerulus responses showing dilation under odor stimulation. Forepaw stimulation induces the capillary constrictions or dilations of the cerebral sensory cortex in rats. Interestingly, it may further activate regenerative events including activity-dependent repair processes in the CNS.

**TABLE 3:** Human astrocyte-based therapeutics and related research.

**Model**	**Cell source and route**	**Measurement**	**Cellular mechanism**	**References**
Rat PD model, female	TH-transduced astrocytic cell line	Improve motor functions	DOPA and DA release	Lundberg et al., 1996
Rat diabetic model, male	hAMSC, intravenous	Improve BRB integrity	Differentiation into GFAP^+^ cells	Yang et al., 2010
Adult immunodeficient mouse	hESC H7 and H9/hiPSC IMR90-4 lines derived S100β^+^/GFAP^+^ cells, intraventricular or intracranial	Form gliovasculature	Differentiation into astrocyte	Krencik et al., 2011
Adult rat SCI model, female	A2B5^–^GFAP^+^ or A2B5^+^PDGFRa^+^Nestin^+^GFAP^+^hGPC, intraspinal	Fill the injury cavity	Differentiation into astrocyte	Jin et al., 2011; Haas et al., 2012
Adult mouse/young adult rat ALS model, female/male	hESC HADC100 and NCL14 lines derived GFAP^+^ cells, intrathecal	Delay disease onset/progression	Neurotrophic factors in the CSF	Izrael et al., 2018
Adult immunodeficient rat, female/male	Nestin^+^hNSC NSI-532 line, spinal subpial	Form GFAP^+^ glia limitans	Differentiation into astrocyte and oligodendrocyte	Marsala et al., 2020

**There has been more research using astrocyte transplantation for ALS or SCI treatment, which is referred to Nicaise et al. (2015).**

A growing body of evidence supports the role of pericytes for LCBF regulation via the NVU and capillary component, in the BBB, and for their interactions with glial cells. During brain hypoxia/ischemia, with constricting capillaries and trapping blood cells in their lumen, pericytes are found to contract and limit LCBF. Hypoxic/ischemia can induce pericytic contraction and cell death demonstrated in the brain and retina explants. Some pericyte subpopulations regulate LCBF dynamics and/or in responses to ischemia and/or hypoxic insults (Yang et al., 2017). There have been reports of subtypes of transitional pericytes with broader distributions, midcapillary pericytes from the capillary bed, and stellate pericytes on postcapillary venules. Additionally, pericyte subtypes may be involved in controlling the BBB permeability over the LCBF (Villasenor et al., 2017). Transitional pericytes may locate on the precapillary and postcapillary structures and express VSMC-associated contractile protein α-SMA independent of astrocytes. Within the brain or retina, we and others have demonstrated the expression of α-SMA in capillary pericytes (Alarcon-Martinez et al., 2018). Midcapillary pericytes show the expression of contractile proteins myosin and vimentin. Interestingly, RACs also re-express the α-SMA and vimentin. Pericyte may have contractility property and is able to contract as well as to dilate capillary structures for the peripheral stimuli and cerebral blood flow controls (Bandopadhyay et al., 2001). Markers for pericytes (e.g., NG2) are not specific, more systematic identification of pericytes will be needed (Smyth et al., 2018). In addition, it is suggested that subtypes of pericytes may show organ-specific features, for example, functioning differently toward specific micro-environment in the brain. In particular, mouse brain pericytes are demonstrated to express contractile proteins such as calponin, desmin, non-muscle myosin variants and skeletal muscle actin, α-SMA, and vimentin by single-cell RNA sequencing analyses (Birbrair et al., 2013).

### Mitochondrion, Ca^2+^, and Ischemia-Reperfusion

The relationship between ATP, energy metabolism, and ischemia has been evidenced from study of mitochondria from ischemic brains showing a decreased ability for the generation of ATPs, which may be correlated to a reduced capacity for cellular respiratory activity and adapted for the cell loss during an acute phase of an ischemia (Kalogeris et al., 2012). Transplantation of cells (e.g., astrocytes, BMSC, NPC, VPC; Supplementary Table S1 for full terms) can be critical sources of activating mitochondrial mechanisms for neuronal protection during neural development as well as in regenerative conditions in NDDs (Spees et al., 2006; Perico et al., 2017).

After ischemic stroke of an adult brain, glucose/glycogen may be rapidly consumed due to aerobic glycolysis for the generation with ATPs and pyruvate while ATPs recovery during ischemia-reperfusion gradually occurs in all the affected tissues but potentially distinguish with ischemia-susceptible and ischemia-resistant regional differences. In general, an ability to provide and maintain relatively high ATPs for cellular activities can be gradually re-obtained. Disrupted mitochondrial functions can be still observed in some ischemia susceptible regions with a delayed reduction of ATPs. Measurements of the energy-related metabolites are important at times and it is developed as useful indicators for mitochondrial activity change and energy metabolic recovery, such that the reduced glucose oxidation from ischemic regions for the first few hours and the neuronal activity-increasing energy need for the post-ischemic brains and an elevation of glucose utilization (Rehncrona et al., 1979). The neurovascular coupling and the ability to recover blood flow to glucose utilization can be significantly varied at this subacute phase and in different brain regions. There are reduced glucose metabolism and low local energy needs within a short period of time, without changing any metabolic capacity at lower blood flow. Meanwhile, continuously reduced needs in ATPs may cause long-term follow-up changes in the brain with acute and chronic hypoxic/ischemic insults. Aerobic glycolytic activity (e.g., pyruvate, catalyzed by PDC) is reduced at the first few hours after ischemic reperfusion. This activity change is usually due to inhibition with reduced glucose oxidation for ischemia. It further inhibits energy producing mitochondrial function at those first few hours of reperfusion. In addition, anaerobic glycolysis then generates lactate from pyruvate and NAD^+^. A level of lactate is shown to be a prognostic value as an outcome of hypoxic-ischemic injury in neonatal brains. Generally, an accumulated oxidative stress can lead to acute cell damage and death, known mainly as the reperfusion injury. Following those first few hours of reperfusion, there are also reductions in an oxidative metabolism, and in energy demands prevailing, which could involve many attenuated metabolic responses, originally evidenced for tissue slices under chemical depolarization stimulation (White and Reynolds, 1996). Those are associated with changes of mitochondria (e.g., failure to respond to increased energy needs). An impairment of mitochondrial function, however, limits the recovery of their normal roles in the cells and the brain tissue. Deficiencies and impairments of mitochondrial functions will lead to chronic deleterious changes and may or may not compromise secondary injuries within ischemia-susceptible regions, contributing to post-stroke depression and neurodegenerative conditions.

There can be changes in pH during ischemia and reperfusion. As in mechanisms of pressurized pial arteries and parenchymal arterioles, the RyRs-mediated Ca^2+^ signals in VSMC provide potential flow responses. Protons (H^+^) can also shape the Ca^2+^ signals in cerebral VSMC. At a physiologic CSF pH of 7.3, pial arteries VSMC show Ca^2+^ sparks, which inside cells are a spatially limited form of Ca^2+^ movement. Alkalosis can occur at higher RyR resting open state probability with Ca^2+^ releases through neighboring RyRs to induce a Ca^2+^ wave. In parenchymal arterioles, however, Ca^2+^ waves are apparent in normal conditions. On the other hand, acidosis can occur at lower RyRs resting open state probability when it can restrict Ca^2+^ sparks. Ca^2+^ signals as well as their reshaping mechanisms are fundamental for acidosis-related vasodilation mechanisms, e.g., acidosis converting Ca^2+^ waves into Ca^2+^ sparks which lead to an activation of BK_*Ca*_ channels. Parenchymal arteriolar VSMC and Ca^2+^ waves, in response to acidosis (for example during an external pH from 7.4 to 7.0), can dilate their parenchymal arterioles, involved in the reshaping mechanisms of predominant intracellular Ca^2+^ (from Ca^2+^ waves to Ca^2+^ sparks) and the activation of BK_*Ca*_ channels. Astrocyte responses in cortical parenchymal arteriolar vascular responses and astrocytic Ca^2+^ dynamics can be measured using an *in vitro* rodent brain slice model of perfused/pressurized parenchymal arterioles; studies have been supplemented with *in vivo* astrocytic Ca^2+^ imaging. Those astrocytes are demonstrated to respond to the flow/pressure increases within the parenchymal arterioles and show subsequent increases of intracellular Ca^2+^ responses. It has also been shown that phenylephrine-caused systemic arterial blood pressure or SBP increases induce astrocytic Ca^2+^ and may be linked to a flow/pressure-induced vasoconstriction response of parenchymal arterioles. Chelation of intracellular Ca^2+^ such as using BAPTA can block the response, together with blocking TRPV4/purinergic receptors on astrocytic surface. These triggering mechanisms seem less effective with K^+^ or 20-HETE signaling blockade (Kim et al., 2015). These will then cause VSMC hyperpolarization/vasodilation. Acidosis within the narrow and physiological pH range induces the vasodilation with remodeling Ca^2+^ signals. Astrocytes are important players in maintaining local brain extracellular pH homeostasis. The data provide evidence of another important metabolic housekeeping function of these glial cells. Under P2Y1/PLC activation, astrocytes may release bicarbonate as a buffering system for extracellular H^+^ against acidification such as when there is an increased neuronal activity (Theparambil et al., 2020). In summary, VSMC of parenchymal arterioles, sensitive to an extracellular pH changing an intracellular pH, Ca^2+^ signals can be greatly affected by the protons (H^+^). Increased CO_2_ from 5 to 15%, together with acidosis effects involving RyR-BK_*Ca*_ channel activation, may cause vascular relaxation. Reduced extracellular pH can lower cytoplasmic Ca^2+^ and the RyRs activity (Hidalgo et al., 2004), together with alterations in cellular metabolism, Ca^2+^ homeostasis, or proinflammatory stimulus as cause of significant detrimental consequences in astrocytes (Table 4). It is also shown that inhibition on VOCCs-mediated Ca^2+^ influx will not affect the inducing/persistent effects under alkalosis conditions of Ca^2+^ waves of pial arteries in the rat cerebral cortex. At pH 7.0, the protons (H^+^) can lower RyRs resting open state probability, thus prevent an activation of neighboring RyRs by local Ca^2+^ releases and the release in forms of Ca^2+^ quark/sparks (Dabertrand et al., 2012). Interestingly, there is evidence for the increased L-VOCCs in RACs demonstrated in cell culture with triggers of ATP, glutamate, LPS, or K^+^ and partially blocked by L-type VOCC inhibitors (Cheli et al., 2016). On the other hand, intracellular acidification and reduced Ca^2+^ may be responsible for the decrease in the resting open state probability of IP3Rs/RyRs to terminate the Ca^2+^ waves in both astrocytes and VSMC. Preventive Ca^2+^ leak mechanisms of the ER/SR may increase the SR luminal Ca^2+^ concentration. Consequently, there will be increased local spark probability at the junctional SR elements. On the other side, an increase of ER/SR Ca^2+^ load activates RyRs and increases frequency of Ca^2+^ spark. For IP3Rs, as a novel mechanism, the luminal Ca^2+^ binding may inhibit an IP3R activity at the luminal Ca^2+^ concentration of 3–10 mM. Outside the range, there is a potentiation of an IP3-induced Ca^2+^ release by luminal Ca^2+^.

**TABLE 4:** Molecular functions of astrocytes based on PPIs with GFAP.

**Aging**	**BBB/NVU**	**Ca^2+^**	**Psychiatric disorder**	**Other**
*AKT1,AURKB,CDK1,CRYAB,DNAJA3,GRB2,KRT3 3B,MBP,PER1,PPP1R9B,PSEN1*	*AQP4,MBP,PECAM1,SLC1A3*	*CALM1,CALM2,CALM3,CTNNB1,HMGB1,KCNMA1,MYO5A,NSMF,PRKCA,PRKCG,RORA,WFS1*	*APP,CTNNB1,DIPK2A,PRNP,SMARCA2*	*ABI2,ABLIM2,AIF1,ALB,ALDH1A1,ALDOC,ALG3,AMELX,ANLN,APLP2,AP2B1,AP2M1,ARFGAP1,ASB3,ASIC4,ATOH1,ATP6V1B2,BAHD1,BAIAP2,BCAS2,BIRC2,BRK1,CARD10,CASP1,CCDC57,CCDC120,CCDC196,CCNC,CDC23,CDC37,CEBPA,CEP76,CFAP206,CHMP4B,CIT,CLEC4M,COL16A1,COQ2,COX6A1,CRLF3,CT55,CWF19L2,C6orf141,DBF4,DCX,DDIT3,DDI2,DES,DNAH14,DNASE1L1,DNM1L,DPF1,ECT2,EGF,ELAPOR1,ENKD1,EPM2AIP1,ERBB3,ERCC8,ERRFI1,ESRRB,ESR2,ESS2,EXTL2,FASTKD1,FAM50B,FANCG,FBXO25,FOXA2,FXR1,FXR2,FYN,GABARAP,GABARAPL1,GABARAPL2,GOLGA2,GPR108,GRAP2,GRB10,HEATR1,HEXIM1,HGS,HK3,HNRNPK,HOMEZ,HOXA9,HSPA5,HSPA8,HTT,H3C15,ICA1L,IFT20,IKBKG,IL16,INA,IQUB,ITM2B,JADE3,JPH3,KIAA0408,KIAA1217,KIFC3,KIF14,KIF20A,KIF23,KLHL20,KRT13,KRT15,KRT19,KRT27,KRT31,KRT39,LASP1,LENG1,LENG8,LGALS9C,LGALS14,LMO2,LNX1,LONRF2,LPIN1,LSM14B,MAPK8IP2,MAPK9,MAPT,MAP1LC3A,MAP1LC3B,MAP2,MCRS1,MEN1,MEPCE,METTL27,MIB1,MICAL2,MKL1,MLC1,MOS,MSRB2,MTF2,MTNR1A,MYBPHL,MYLPF,MYO15B,NAA16,NAP1L3,NCOR1,NEBL,NEFL,NEFM,NEK6,NES,NFKBID,NFX1,NHLH2,NME2P1,NOD1,NPHP4,NSF,NTAQ1,NT5DC1,NUDT21,NXF1,ODF3L2,OLIG2,OTUB1,OTUD7B,PCSK7,PDCD6IP,PDLIM1,PDLIM5,PDLIM7,PDZK1,PERP,PHF19,PIAS2,PIH1D2,PIK3R1,PKP3,PLB1,PLP1,PNMA5,PNP,POM121,POU5F1,PPP1R16A,PPP1R16B,PPP1R18,PPP1R21,PSEN2,PTBP3,PTPRT,P2RY8,RAB3IP,RAB5A,RAB38,RAD23B,RASSF1,RASSF2,RASSF5,RASSF8,RBFOX3,RECQL4,RHPN1,RIBC2,RSPRY1,RUFY4,RYBP,SAMD3,SAXO1,SCAPER,SHC1,SH3YL1,SKP2,SLU7,SMAD2,SMARCB1,SMARCD1,SNRNP40,SNW1,SOAT1,SPATA2,SPATA2L,SPATA22,SPDL1,SPRED1,SPRED2,SRC,STAT1,STK36,SULT4A1,SYNC,SYP,SYT2,S100B,TAB2,TARDBP,TASOR2,TBC1D21,TBC1D22B,TCAP,TEKT2,TFCP2,TFIP11,THAP1,TLE5,TLR4,TMEM186,TNFAIP1,TOM1,TPT1,TP53BP2,TRIM27,TRIM69,TSC1,TSNAX,TUBB3,TUBGCP4,TUT7,TXN,UBASH3A,UBASH3B,UBC,UBE2I,UBE3A,UBR1,UBR2,UBXN8,USP10,VIM,VSX2,WDR61,WDR83,WDYHV1,WWOX,YAP1,YES1,YWHAE,YWHAZ,ZC2HC1C,ZFPM2,ZHX3,ZKSCAN3,ZKSCAN5,ZNF19,ZNF20,ZNF232,ZNF302,ZNF410,ZNF436,ZNF444,ZNF488,ZNF500,ZNF572,ZNF581,ZNF655,ZNF774,ZSCAN9,ZSCAN22,ZUP1*

*Protein-protein interactions with the reactive astrocyte specific marker GFAP were based on data from human/mouse/rat homologous proteins. According to protein annotation databases and our previous publication (Zhang et al., 2020), some genes potentially related to severe mental illnesses (SMI) or other psychiatric disorders, and Ca^2+^-dependent proteins are summarized. Considering that schizophrenia and some other neuropsychiatric problems such as autism may share common traits, we included some of the other neuropsychiatric disorders whose genes are the primary causes, and which are relatively mild-to-moderate. Other potential genes and categories (including aging related genes, and genes of the BBB/the NVU) were identified in the UniProtKB protein annotation/knowledge database.*

Ion channel regulation of mitochondrial Ca^2+^ influx and efflux is known to regulate the mitochondrial responses during hypoxia/ischemia and in conditions of NDDs. Since both the extracellular Ca^2+^ and Ca^2+^ released from the ER/SR determine mitochondrial Ca^2+^ levels, an increased mitochondrial Ca^2+^ is usually associated with increased mitochondrial matrix enzymatic activities and with augmented ATP production (Tarasov et al., 2012). However, excess Ca^2+^ entries in astrocytic cells and neurons following mitochondrial dysfunction of ischemic reperfusion can be observed (Cross et al., 2010; Belov Kirdajova et al., 2020). This may trigger neuronal necrosis in acute phases of ischemic stroke and cause astrogliosis in a subacute and chronic conditions of NDDs. Ca^2+^ movement from the ER/SR-to-mitochondria occurs at the specialized contact sites of mitochondrial-associated ER membranes. Ca^2+^ movement is therefore pushed into the mitochondrial matrix through IP3Rs, VDACs in the outer mitochondrial membrane, and the uniporter complex, and is responsible for the pathological changes in both acute and chronic processes. Mitochondrial Na^+^/Ca^2+^ exchanger (human gene name: *SLC8B1*) is mainly responsive for Ca^2+^ removal, which may be also dependent on intracellular Na^+^ beyond threshold, especially in conditions of energy deficiency (Dipla et al., 1999). A family of BK_*Ca*_ channels has been suggested as a promising neuroprotective target, for protective attenuation of Ca^2+^ influx and ROS production in mitochondria upon membrane depolarization and/or intracellular Ca^2+^ accumulation (Jacobsen et al., 2018). Dysregulation of SLC8B1 has been reported to be linked with pathophysiological progression of AD.

On the behalf of mitochondria-dependent cell death, a key component, the mPTP subside, which is part of mitochondrial Ca^2+^ homeostasis for normal conditions; when it prolongs opening, it leads to apoptotic and necrotic cell death under hypoxia/ischemia and many other pathological conditions. The releases of AIFs, cytochrome c, endonuclease G, Smac/DIABLO into the cytoplasm activates apoptotic cascades, especially in the ischemia-sensitive neurons of the hippocampus CA1 area, can be the primary cause of the neuronal loss. Ca^2+^, H^+^, ROS in overload interact with polyphosphates (polyPs) of the inner mitochondrial membrane to cause the mPTP opening. Other related cellular and pathobiological processes may include membrane potential dissipation leading to impaired respiratory chain, halted ATP synthesis, organelle swelling, and outer membrane rupture. PolyPs, the polymers of linear, cyclic, or branched, with large negative charges, and the ubiquitous metabolites, are potentially interacted with many mitochondrial proteins, for their mPTPs being regulated through protein binding activities (Wei et al., 2015). *In vivo* studies have shown that the astrocytic releases of polyPs can evoke Ca^2+^ signals and mitochondrial deletion of polyPs can reduce mitochondrial membrane potential and inhibit the Ca^2+^-induced mPTPs opening. It is considered as a protective condition when there are depolarized mitochondrial membrane potentials as well as delayed opening of mPTPs. Exogenous cells may potentially protect from an ischemic injury through mPTP blockage of endogenous cells. mPTP is also widely involved in chronic diseases and tumor progression (Patki et al., 2009; Bonora and Pinton, 2014; Kalani et al., 2018). It has been believed that induced permeabilization of the outer mitochondrial membrane can activate phagophore formation of autophagy pathways or initiate irreversible cell death pathways, which involve BAX/BAK and mPTP mechanisms. On the other hand, mitochondrial autophagy, or mitophagy, can promote mitochondria turnover and clear dysfunctional mitochondria. Hypoxia/ischemia activate AMPK/mTOR axis, Bcl-2/BNIP3, FoxO3, HIF-1α, NF-κB (Supplementary Table S1, for full term), together with signals of ER stress and oxidative stress, for mitophagy and an elimination of an affected mitochondrion. The selective process involves fission complex and degradation of the dysfunctional mitochondrial fragment. PINK1 (Parkinson disease associated gene) and Parkin in between mitochondrial membranes are natural regulators of mitophagy (Geisler et al., 2010). Like mechanisms under hypothermia, more research is ongoing to show that the cell therapy can reduce Parkin mediated mitophagy and autophagic cell death. In our recent research, pharmacological hyperthermia that is developed by our group (Lee J. et al., 2016) promptly protects the cells of the NVU and capillary component following transient middle cerebral artery occlusion model of the mice (Zhao et al., 2020).

Mitochondrial transfer and biogenesis promoted by transplanted BMSC may be beneficial mechanisms at an acute phase for a neuroprotection strategy. Importantly, a mitochondrial transfer through connexion–43 (Cx−43)-containing gap junctional channels has been demonstrated in rodent airway and lung injury models (Yao et al., 2018). Endogenously, transfer of mitochondria may be provided from astrocytes to neurons after stroke (Berridge et al., 2016; Pluchino et al., 2016). Mitochondrial fission and fusion processes, central events of mitochondrial biogenesis, may be controlled by several transcriptional factors, such as NRF-1 and PGC-1α. Hypoxia/ischemic insults may inhibit the mitochondrial biogenesis (Zhang et al., 2007). As invested for both acute and chronic regimen, cell transplantation therapy should also provide corrections for energy downshift during acute hypoxia/ischemia and chronically progressed NDDs. For the cells better reaching the brain regions, research has suggested that intranasally delivered cells demonstrate the ability to cross the cribriform plate connecting to the nasal cavity to the olfactory bulb of the forebrain but remained outside of nerve tracts at an early time point of 2 h post administration (Galeano et al., 2018). As another important endogenous mechanism, continuous biogenesis of mitochondria will improve energy production capacity for clinically relevant neuroprotective strategies. This needs to be addressed in future research. Glial cells and NSC/NPC both express Cx-43 and demonstrate active mitochondrial transfer mechanisms. Transplanted subtypes of glial cells and NSC/NPC directed toward terminal differentiation, however, greatly exhibit potentials in providing multiple benefits for host cell populations, in addition to cellular/subcellular replacement/donation strategies.

### The NVU, Ca^2+^, Pericyte, and Energy Metabolism

Astrocytes can modulate neuronal activities, which are primarily involved in mechanisms at levels of capillary regulation for LCBF. In addition to effects of neuronal connections, neuronal ATP binds to astrocytic P2X, which activate PLD2-diacylglycerol-AA and diacylglycerol lipase. COX1, together with prostaglandin PGH2/PGES, mediates AA-prostaglandin PGE2 production. Prostaglandin PGE2 will then activate pericytic prostaglandin PGE2 EP4R receptor and cause pericytic relaxation. Astrocytes can produce AA for pericytes to induce pericytic depolarization/contraction controlled by cytochrome P450-mediated 20-HETE production inside the pericytes. Within the reach of the NVU and capillary component, neuronal releases of glutamate can activate astrocytic mGluRs for a subsequent intracellular Ca^2+^ concentration increase in an IP3-dependent manner and thereby induce the PLA2-AA pathway (Wang et al., 2009). Similarly, releases from neurons, astrocytic adenosine, ATPs, or noradrenaline can affect the pericyte contractile state, such that adenosine binding to α1/2-ARs, together with activated K_*ATP*_ channels, cause pericytic hyperpolarization/relaxation. An activation of many K^+^ channels including BK_*Ca*_/IK_*Ca*_/SK_*Ca*_ and KIR channels will cause pericytic hyperpolarization and decrease their intracellular Ca^2+^ influx. Neuronal NO toward pericytes will inhibit their AA-related 20-HETE production and cause pericytic relaxation. Regarding arteriolar regulation of cerebral blood flow, one of the mechanisms behind functional hyperemia is the regulation of NOS and the production of NO in both astrocytes and neurons which promote VSMC hyperpolarization/relaxation, supporting the role of NO in capillary dilation. Noradrenaline/α2-ARs signaling increases intracellular Ca^2+^ and causes depolarization/contraction. Similarly, to endothelial cells, ATPs activate pericytic P2X/P2Y (such as P2X_7_ and P2Y_4_) for depolarization, increased intracellular Ca^2+^, and cause pericytic contraction. Neuronal K^+^ release and reduced K^+^ import largely increase extracellular K^+^ and activate L-type VOCCs on the pericyte surface with increased pericytic intracellular Ca^2+^ and depolarization/contraction. Endothelial endothelin 1, PDGF-B, and other vasoconstrictors, as well as the ETAR/IGF1R/PDGFR-β signaling, also cause pericytic depolarization via Ca^2+^ influx (Kawamura et al., 2004). The NVU and capillary component for these small vessels in the brain, where they branch off from connecting precapillary arterioles, enrich microvascular network of as large as 120 cm^2^/g in surface area for the brain available in transporting exchanges of molecules across the endothelium. Brain capillary pericytes are located in the basement membrane of endothelium (Thomsen et al., 2017). Together with endothelial cells, pericytes are presented with stretchable processes along and around capillary structures (including the connecting precapillary arterioles and the postcapillary venules). Differentially, they tend to be apparent in more longitudinal processes at the capillary bed of capillary network, in more circumferential processes within the precapillary arteriole, and in more stellate morphology at the postcapillary venule. Endothelial cell-pericyte junctional interactions occur as the expression of connexins (such as Cx37 and Cx43) in pericytes verified by a single-cell RNA sequencing study of the mouse cortex and hippocampus. Pericytes have been shown to promote vasculogenesis, collaterogenesis, and angiogenesis during development of the brain, become part of the BBB, and as functional compartments, be involved in interacting with stem cells, neuroinflammatory actions, and toxins clearance (Sweeney and Foldes, 2018). Mature pericytes then become LCBF regulators and by regulating the diameter of capillary vessels and the metabolic and Ca^2+^ signals, actively involve the capillary vascular tone (Cai et al., 2018). Pericyte of both endogenous and exogenous sources may interact with NSC or co-transplanted cells to promote flow recovery (Sun et al., 2020).

At earlier studies on neurovascular coupling, it is realized that the NVU and capillary component regulates LCBF of the brain for the ability and mechanisms of functional hyperemia, where the coordination between neural activation state and LCBF has laterally been recognized (Nippert et al., 2018). With metabolic needs, the regional increases in LCBF are observed, delivering more substrate at local levels of the NVU and capillary component. Those critical mechanisms linking between neural metabolism and cerebral perfusion, can be affected by, or even lead to progressive impairment of the brain structure and function in NDDs, evidenced by anatomic and functional studies and functional hyperemia observations. Upon neuronal activation and circuit activities, parenchymal arterioles of the higher vascular resistance and rapid dilation capacity within the cerebral cortex are main contributors to the LCBF regulation where there is intracellular Ca^2+^ in VSMC of pial arteries/penetrating arterioles. It is convinced that the intracerebral microcirculation can actively and substantially regulate LCBF and global flow. Since the NVU and capillary component could be formed from an astrocyte and its end feet, an endothelial cell, a microglia, a neuron and its terminals, an oligodendrocyte, a pericyte or even a VSMC (Brown et al., 2019), it needs to be involved in contractile phenotype of the vessel wall altogether to integratively control the vessel diameter. Cell typic composition may vary depending on the branching levels, locations, and sizes. On the neuronal mechanisms of flow controlling, dilation of arterioles depends on an activation of NMDARs on the cell surface and Ca^2+^-dependent NO production by the pre-existing interneurons (Kisler et al., 2017a). Additionally, synaptic release of neurotransmitters, particularly the excitatory signaling pathway within the brain using glutamate, can activate mGluRs on both descending neurons and surrounding astrocytes. Altogether, it can further generate cytoplasmic Ca^2+^ increases, followed by the propagated activation of IP3Rs/Ca^2+^ to reach the physiologically elevated Ca^2+^ and related signal levels at an astrocytic end feet, which have been maintained at normal levels, as astrocytic Ca^2+^ increases in response to sensory stimulus. Since there is evidence supporting a unit of the NVU and capillary component as an integrated functional module, with two major sorts: types of neural and vascular cells; neurovascular dysfunction might occur in NDDs diseased state and cause microvascular detrimental changes. In addition, there could be acute and chronic arterial and arteriole damages leading to significant reduction/less regulation of blood flow or even serious vascular events. Examples have included CAAs and some other cerebrovascular diseases. Transplanted cells seem able to repair vasculature damages as evidence shows co-transplantation of vascularization supporting cells or materials improving engraftment (McCrary et al., 2020). Extensive research focused on vascularization following transplantation has revealed included mechanisms of *a*FGF/*b*FGF and other FGF signaling, PDGF signaling, and VEGF signaling. As a complementary mechanism in astrocytes, the non-selective TRPV4 receptor can mediate Ca^2+^-induced Ca^2+^ release at the astrocytic end feet, to potentially amplify a neurovascular coupling followed by an activation of BK_*Ca*_ channels and K^+^ release into an extracellular space. The moderately increased concentration of extracellular K^+^ can activate KIR channels in VSMC causing their hyperpolarization/relaxation (Jackson, 2017). TRPV4 mutations have been associated with many developmental disorders such as types of neuromuscular and MND diseases. The pathophysiologically high extracellular K^+^ levels, such as in spreading depolarization of the cortex, activate VOCCs in VSMC and cause VSMC depolarization/contraction. Since there are higher correlations of the tissue metabolic state and arteriolar dilation/constriction, such that on brain slices at a partial pressure of 20% O_2_, levels of lactate are higher than that at 95% O_2_. The level of prostaglandin PGE2 can be affected by extracellular lactate, while blockage of a prostaglandin PGE2 reuptake can be managed on the functional activity of prostaglandin transporter (PGT) (Chan et al., 2002). Inhibition of prostaglandin PGE2 reuptake by astrocytic surface PGT increases prostaglandin PGE2 accumulated at the extracellular spaces for VSMC relaxation. Activity-induced physiological adenosine and ATPs will directly cause VSMC constriction after activating P2X/P2Y or VSMC relaxation via A2ARs activation. That extracellular K^+^ concentration shows activated VSMC-related VOCCs and VSMC depolarization/contraction mediated through intracellular Ca^2+^, involving astrocytes during the neurovascular coupling into arterioles (Horiuchi et al., 2002). Typically, between neurons and astrocytes, neuronal ATPs and glutamate transmitters release may be able to activate astrocytic P2Y and mGluRs for IP3-dependent intracellular Ca^2+^ increase, which has been shown in some studies to contribute to mechanisms of neurovascular coupling. Intracellular Ca^2+^ rises-activated signaling cascades in astrocytes also include vasoactive release/efflux at the end feet connecting to VSMC, such as BK_*Ca*_ channel mediated K^+^, PLA2-AA-EETs, cytochrome P450 and COX1-related prostaglandin PGE2. On the other hand, TRPV4 facilitates extracellular Ca^2+^ intake for an intracellular Ca^2+^ increase in astrocytes and pericytes (Rakers et al., 2017). There are lack of, however, lines of evidence for exact molecular pathways being involved. Most research to date, revealed largely increased extracellular K^+^, as described above, will likely activate the voltage dependent VOCCs linked to increased intracellular Ca^2+^ and induce VSMC depolarization/contraction during those physiological processes. VSMC prostaglandin PGE2 EP4R receptor activation increases cAMP, then related to the induction of hyperpolarization/relaxation (Hristovska et al., 2007).

Lactate (formerly introduced) as an aerobic metabolite of glycolysis is presented in astrocytes as well as in transplanted cells. The released extracellular lactate will affect the level of prostaglandin PGE2 and may be also modulable through blockage of prostaglandin PGE2 reuptake by PGT. Lactate regulation is highly related to tissue O_2_ in body homeostasis controls and diseased conditions. In a detailed observatory, AA converted to 20-HETE in VSMC potentially depolarizes the cells and causes VSMC contraction, when especially in high pO_2_ the AA-converted 20-HETE causes arteriolar constriction. Local O_2_ as one of modulatory factors for LCBF, demonstrated by means of brain slices recording and *ex vivo* retina explants and for hyperoxic conditions, can therefore be contributed to regional controls during acute stroke and chronic hypoxic/ischemic conditions. In addition, the effects of lactate on modifying vessel dilation have been demonstrated in miniature pig retina and guinea pig cochlear organ, both showing vasodilation after the systemic or the local lactate administration.

Although astrocytes to VSMC communications via the molecular pathways have been also suggested and partially evidenced, there may be still a controversial view to an astrocyte involvement in the neurovascular coupling connecting to the end and smallest arteries, different from the NVU and capillary component. So far, evidence-based rationale for those neurovascular couplings may have included: *a*. astrocytic modification on synaptic activity and neuronal modification on astrocytes; *b*. differences of astrocytes between them and arteriolar or capillary structures; *c*. chemical and physical communications among astrocytes, pericytes, and VSMC (Sweeney et al., 2016). Normally, endothelial, or neuronal NO release blocks 20-HETE production by VSMC and can involve the sGC-mediated cGMP mechanisms to cause VSMC relaxation (Carvajal et al., 2000). Astrocytic Ca^2+^ responses, in COX-dependent pathways, or specification for RACs may also induce eNOS activity, suppress 20-HETE levels, and facilitate PGE2-mediated vasodilation. Neuronal adenosine release can act on A2AR activations to block VSMC VOCCs activation, for inducing VSMC hyperpolarization and relaxation (Murphy et al., 2003). In the brain, adenosine release and A2AR may be considered as one of the triggers to neurodegeneration. Pre-AD conditions such as in aging mice with human Aβ deposition can increase astrocytic A2AR. Like types of gliotransmitters such as glutamate and D-serine, RACs may also release ATPs via vesicular exocytosis; ATPs leakage is also observed through hemichannels. In addition, A2AR on microglia may be involved in potentiation of NO release. ATPs released from neurons also activate P2X/P2Y receptors on VSMC followed by increased Ca^2+^ influx, membrane depolarization, and contraction (Mulryan et al., 2000; Ho et al., 2015). Vasoactive releases of acetylcholine/bradykinin, ADPs/ATPs, UTPs from the blood stream and cooperatively activated acetylcholine receptor muscarinic M3, bradykinin receptor B2 and P2X/P2Y receptors initiate the signaling of astrocytes and endothelial cells to transmit downstream signaling cascades (Hösli and Hösli, 1993). The diacylglycerol-AA pathway and PLC activation can act on the VSMC through vasoactive releases. In the activation of receptor-mediated signaling and increased intracellular Ca^2+^, endothelial cells will produce and release AA, EETs, or prostacyclin PGI2. Endothelial AA, EETs, and prostacyclin PGI2, similarly to prostaglandin PGE2, causes VSMC hyperpolarization/relaxation. In general, increased Ca^2+^ is shown to activate eNOS and NO production, and to release K^+^ via K_*Ca*_ channels from endothelial cells, causing VSMC and endothelium hyperpolarization. Normally, astrocytes are critical in the control of K^+^ homeostasis by clearing the extracellular K^+^ and thereby constantly involved. In blood vessels and flow regulation, VSMC hyperpolarization is also triggered by EDHFs while activation of endothelial eNOS and AA-related pathways are extracellular ATP-dependent in response to endothelial vessel wall change and RBC under the shear stresses. Transplantation of astrocyte may enhance the intercellular communications (Lepore et al., 2008). These may include enhanced responsive vasculature integrity or the ISF clearance mechanisms. There are gap junctional functions and involvement between endothelial cell-VSMC and in endothelium, reported to mediate the endothelial responses and related signaling toward VSMC for endothelium-mediated regulation of VSMC tone. Endothelial cells can modulate vascular tone in forms of vasodilation. In rodent tissues, it is evidenced that the hyperpolarization of endothelial cells will travel no shorter than 1 mm within the endothelium, where the related dilation can be mediated by endothelial cell to VSMC signal, or myoendothelial coupling through gap junctions or via EDHFs (Takano et al., 2004).

It has been described that the role of RACs is important, but still it may not be possible to provide a detailed description of RACs; additionally, whether the role of RACs in stroke is similar with NDDs has yet been unknown. It has been suggested that astrocytes may act as bidirectional sensors (including endothelial cells and neuronal involvement) and send adjustments with brain perfusion, basal hemodynamics, and parenchymal arteriolar tone. Examples of the neuronal involvement in the regulations of the VSMC tone have included that an activation of neuronal NMDARs has dose dependent dilation effects in parenchymal arterioles. NMDA/other chemical/optogenetics stimulating neuronal excitability (such as of the striatum from newborn sheep, or the rat somatosensory cortex), and showing increased LCBF, supports the critical roles for the release of neuronal NO in regulation of LCBF (Estrada and DeFelipe, 1991). Neuronal adenosine has an endothelium-independent vasodilator effect for VSMC relaxation. Vascular controller of neural modulating origins has included vasoactive intestinal polypeptide, dopamine, substance P, serotonin, γ-aminobutyric acid, noradrenaline, neuropeptide Y, somatostatin, and acetylcholine. Coordinately, different types of interneurons have been suggested to control LCBF responses, possibly through astrocytes/pericytes. Animal studies support that acetylcholine releases from cholinergic afferents may promote LCBF; and that noradrenaline releases from locus coeruleus afferents cause vasoconstriction. Evidenced by optogenetic multiphoton imaging, excitatory as well as inhibitory neuronal activities are demonstrated to signal arteriolar dilation. Neuropeptide Y can cause arteriolar constriction.

As described, Ca^2+^ influx in astrocytes may be able to regulate the arteriolar responses. Astrocytic Ca^2+^ are involved in regulation of the arteriolar vascular tone, suggested by previous research showing that there is an increase in intracellular Ca^2+^ into astrocytes changing an arteriolar constriction or dilation. Evidence in astrocytic IP3R2 knockout mice have suggested an impaired Ca^2+^ release or storing can be related to persist arteriolar dilation following stimuli triggers. So far, the GPCRs-IP3R-Ca^2+^ have been demonstrated in astrocytes coupling with subtypes of mGluRs and purinergic receptors (e.g., P1 receptors and several subtypes of P2 receptors). Astrocytic mGluR3 seems not to serve as one of the triggering factors to induce Ca^2+^ response that directly relates to the flows. In some astrocyte populations, ATPs are probably via purinergic receptors for related cell signals being transmitted to the arteriolar tone but not through neurovascular coupling to arterioles and VSMC. Interestingly, astrocytic end feet Ca^2+^ generally precedes the VSMC depolarization/contraction and an arteriolar constriction, which may involve the phospholipase PLA2-dependent pathway and metabolic control from cytochrome P450 in these cells. Other pathways include mGluRs and the COX1-mediated prostaglandin PGE2-dependent pathway after whose activation intracellular Ca^2+^ in astrocytes leads to VSMC relaxation and the dilation of arteriolar structures. Demonstrated in the somatosensory cortex, the photolysis of caged Ca^2+^ can be imaged in astrocytic end feet which sheath the end and smallest arteries and arterioles altogether leading to vasodilation. The involved mechanisms have been related to COX1, cytochrome P450, prostaglandin PGE2-cAMP, and PLA2-AA-EETs pathway in and released from astrocytes (Ding et al., 2020). EETs can hyperpolarize the VSMC membrane and relax VSMC via increased extracellular K^+^ through BK_*Ca*_ and KIR channels. The cAMP mediates VSMC hyperpolarization and relaxation. Utilizing GECIs, it can be shown that physiological neuronal activities should increase Ca^2+^ influx into astrocytic processes around neuronal terminals, linked to LCBF mechanisms at the capillary and regulation of the flow, LCBF, and neurovascular coupling processes. Interestingly, capillary astrocyte and astrocytic Ca^2+^ may play a role together in functional hyperemia, with potential interactions of capillary pericytes or VSMC via neurovascular coupling within arterioles. On the other hand, a persistent increase in pericytes during spreading depolarization is related to Ca^2+^ prolonged vasoconstriction; constriction of cortical capillaries where Ca^2+^ changes of pericytes at first order capillaries significantly regulate LCBF is determinant to spreading depolarization and desensitization to somatosensory stimulation. Consistently, it is demonstrated that desensitized response can no longer evoke changes in either capillary diameter or pericyte Ca^2+^.

### Perivascular Mechanisms in NDDs

Cerebrovascular changes/events in the form of chronic lobar intracerebral hemorrhage, microbleeds and superficial siderosis of hemosiderin deposition may contribute to the severity of NDDs by affecting vasculature functions and perivascular drainage. Microglia/macrophages are potential well mechanism candidates and drug targets (briefly shown in Figure 1; Lee J. H. et al., 2016), which is not the primary focus of this review. Perivascular drainage has been considered as a major route of Aβ clearance from different brain regions (Diem et al., 2018). Accumulation of Aβ as plaques in artery/vain walls as CAA is associated with failure of perivascular elimination of Aβ from the brain ISF in the elderly and in AD. Aβ has been demonstrated as pathological changes in AMD, and some other NDDs. The failure of perivascular drainage of solutes from the CAA brain leads to loss of homeostasis for the neuronal microenvironment (Weller et al., 2009). Therefore, CAA can contribute to the AD etiopathogenesis by directly affecting perivascular drainage. Clinical data suggest that the presence of advanced CAA in AD is associated with greater cognitive impairment and/or faster cognitive decline. Endogenous pathways that are known to be involved in clearing soluble Aβ from the brain have included transport across the BBB, enzymatic degradation, perivascular drainage, and phagocytosis. There are some potential roles of CAAs-related pathology in the development of imaging abnormality ARIA that have been identified as major adverse effects of anti-Aβ immunotherapy (Martínez-Lizana et al., 2015). In reports on CAA patients, in response to visual stimulation, there is reduced evoked vascular reactivity, including that based on the BOLD signal in the visual cortex. Cell therapy will likely help drainage of Aβ along perivascular pathways in aging artery walls to improve cognitive function in synergy with combinatorial approaches such as immunotherapy. Following AD pathology present in around two third of brains from patients with cognitive impairments, significant vascular pathologies were prevalent as risk factors including gross ischemic infarcts, moderate-to-severe CAA, atherosclerosis, and arteriolosclerosis. Each of them was present in up to one third of the patients’ brains. Regarding AD pathologies, precise mechanism of AD-related brain injury remains unclear, however, Aβ-triggered synaptic/neuronal loss seems to be at the central part of AD pathologies. However, among CAA-related brain injuries, the anti-Aβ passive immunotherapy causing additional brain inflammation and the BBB disruption can lead to brain edema (appeared as ARIA-E). Damages of the BBB and microvascular structure further lead to hemosiderin deposition within different areas of the brain (appeared as ARIA-H) (Shih et al., 2018). On the other hand, an ischemic form of CAA-related brain injury may show up as white matter hyperintensities on T2-weighted MRI, structural disconnection measured with diffusion MRI or diffusion tensor imaging (DTI), and cerebral microinfarcts. In ischemic stroke patients, cerebral microbleeds are very commonly presented while it occurs at 1/6–1/4 in those receiving intravenous recombinant tissue plasminogen activator (rtPA). Moreover, although inflammation itself does not seem to be a major component in CAA-related microhemorrhage or microinfarction, cell transplantation is a potential player in modulating immunoinflammatory reactions, in addition to the primary expectations on vascular repair by the cells.

Aβ removal of antibody fragment methods uses the administration of laboratory-produced antibodies to Aβ. Based on the theory of “the amyloid hypothesis,” which highlights flaws in the processes governing production, accumulation, or disposal of Aβ as a primary cause of Alzheimer’s. The recently developed antibody mediated Aβ clearance has demonstrated the ability to clean the small, soluble aggregates of Aβ that are demonstrated to be more toxic. A few strategies to block the effects of Aβ have shown greater benefits in animal models and reached human clinical trials. So far, there is no clear indication that removing Aβ via antibodies or antibody-originated fragments can moderate Alzheimer’s brain changes or protect affected brain regions, or no significant association with the Aβ clearance. A group of patients treated with Aducanumab, which removes both insoluble and soluble forms of Aβ in the brain, have shown a slower progressive rate of cognitive decline following a diagnosis of mild or preclinical Alzheimer’s disease. Aβ bound to IgG is cleared via the Fc receptor-dependent uptake pathway by phagocytic cells including activated microglial cells, which also release inflammatory mediators and neurotoxic factors. Moreover, some Aβ-binding monoclonal IgGs which have demonstrated the ability to clear parenchymal Aβ_42_ also increase Aβ_40_ deposition in blood vessel walls. This can further lead to microhemorrhages, CAAs, and cognitive impairments that correlate the vascular damages. The removal of deposits, however, may cause additional vascular changes, which can have good and bad impacts chronically in aging brains. Stem cells including glial cells, will have the potential to protect the vasculature-related changes.

Some recent progresses of related antibody therapies may include: the catalytic immunoglobulin V domain (IgV) degrades and clears Aβ specifically with no evidence of microglial activation or microhemorrhages. Many monoclonal hAβ antibodies such as Bapineuzumab IgG1 and the single-chain variable fragment (scFv) fusion proteins such as scFv-h3D6 (derived from the IgG-h3D6) are effective in eliminating the most toxic species of hAβ-oligomers (üell-Bosch et al., 2020). The scFv is considered to clean without triggering Fc-mediated effector functions. The scFv-22C4 derived from the IgG-22C4 against C-terminal hAβ is shown to predominantly bind monomeric hAβ_1–4__2_, and inhibit hAβ_1–4__2_ aggregation (Cattepoel et al., 2011). It needs to be confirmed whether vascular repair mechanisms will benefit any treatments saving neurons.

## Conclusion

The overviewed literature provides evidence that hypoxia/ischemia in stroke and/or other similar/dissimilar chronic NDDs conditions can cause parallel changes in mitochondrial activity and ATP production. The initial ischemic reperfusion or adaptive changes recover energy-related metabolites and mitochondrial function but activate Ca^2+^ signaling and some other detrimental mechanisms. Ca^2+^ movement from cytoplasm and the ER/SR into mitochondria and dynamic alteration of oxidative glucose metabolism restrict the respiratory capacity and lead to chronic cytotoxic effects or delayed cell death. The mitochondrial dysfunction contributes to neurodegenerative processes during development or aging and after hypoxia/ischemia, making mitochondrial homeostasis a potential target for pharmacological and cell-based therapies. As part of the flow recovery mechanisms, in VSMC Ca^2+^ sparks, RyRs in regulation of the vascular function have been convincedly identified. At pressurized arterioles, the Ca^2+^ waves in VSMC are tightly controlled by RyRs-mediated Ca^2+^ releases. Following Ca^2+^ waves and persistent chronic changes, there are Ca^2+^-dependent gene activation, such as in astrocytes triggered gliosis and GFAP involvement. Acidification may induce Ca^2+^ spark where the activation of RyRs and BK_*Ca*_ channels provides the novel mechanism as a vasodilator for enhanced LCBF, but it may be related to pathological changes in NDDs. RyRs mechanisms with the ER important for regulation of the flow, LCBF, and neurovascular coupling processes, as top vascular tone regulators within brain parenchymal arterioles may be critical to the neurovascular coupling/uncoupling in both physiological conditions during development and pathological changes in NDDs. Transplantation of astrocyte, or via modulation of different types of glial cells, can enhance mitochondrial functions, vascular repair, and functional recovery for NDDs.

## Author Contributions

YiZ, XS, and ZW wrote the first draft of the manuscript. JY, DQ, XG, MY, YaZ, ZL, YoZ, and LW edited the manuscript. SW, ZH, and ZW wrote the manuscript. All authors contributed to the article and approved the submitted version.

## Conflict of Interest

The authors declare that the research was conducted in the absence of any commercial or financial relationships that could be construed as a potential conflict of interest.
