# Supplementary figures and images for: Glial Cell-Based Vascular Mechanisms and Transplantation Therapies in Brain Vessel and Neurodegenerative Diseases
Source: Front Cell Neurosci. 2021 Mar 26;15:627682. doi: 10.3389/fncel.2021.627682 (PMC8032950; doi:10.3389/fncel.2021.627682)

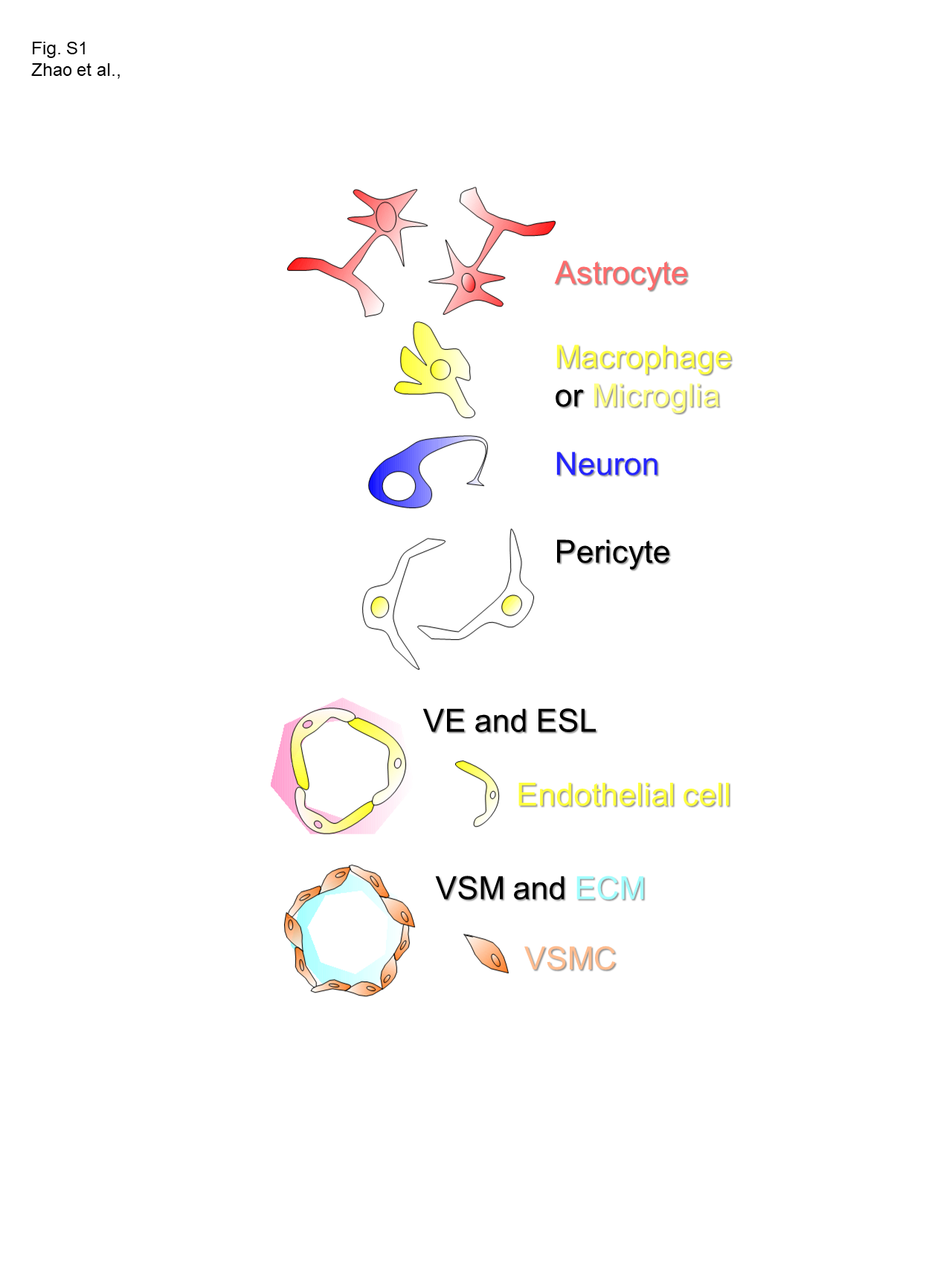

Supplement: Supplementary file 1 [file Image_1.tif]
