# Supplementary material for: Glial Cell-Based Vascular Mechanisms and Transplantation Therapies in Brain Vessel and Neurodegenerative Diseases
Source: Front Cell Neurosci. 2021 Mar 26;15:627682. doi: 10.3389/fncel.2021.627682 (PMC8032950; doi:10.3389/fncel.2021.627682)
Supplement: Supplementary file 2 [file Table_1.DOCX]

| **Short term** | **Full term in the manuscript** |
| --- | --- |
| AA | arachidonic acid |
| Aβ | beta-amyloid |
| AD | Alzheimer's disease |
| ADP | adenosine diphosphate |
| *a*FGF | acidic fibroblast growth factor |
| AIF | apoptosis inducing factor |
| ALS | amyotrophic lateral sclerosis |
| AMD | age related macular degeneration |
| AMPK | AMP-activated protein kinase |
| AMSC | adipose-derived mesenchymal stromal cells |
| α1/2-AR | alpha1/2-adrenergic receptor |
| ARIA | amyloid-related imaging abnormalities |
| ATP | adenosine triphosphate |
| A2AR | adenosine A2A receptor |
| BBB | the blood-brain barrier |
| BAK | Bcl-2 homologous antagonist/killer |
| BAX | Bcl-2-associated X protein |
| bFGF | basic fibroblast growth factor |
| BK_Ca_ | large-conductance Ca^2+^-activated K^+^ channel |
| BMSC | bone marrow-derived multipotent mesenchymal stromal cells |
| BNIP3 | Bcl-2 nineteen kilodalton interacting protein |
| BOLD | the blood oxygen level-dependent |
| BRB | the blood-retinal barrier |
| CAA | cerebral amyloid angiopathy |
| cAMP | cyclic adenosine monophosphate |
| cGMP | cyclic guanosine monophosphate |
| CNS | the central nervous system |
| CO | carbon monoxide |
| CO_2_ | carbon dioxide |
| COX1 | cyclooxygenase 1 |
| CSF | cerebro spinal ﬂuid |
| CT | computed tomography |
| CTA | computed tomography angiography |
| DSA | digital subtraction angiography |
| DTI | diffusion tensor imaging |
| DWI | diffusion weighted imaging |
| EDHF | endothelium-derived hyperpolarizing factor |
| EET | epoxyeicosatrienoic acid |
| eNOS | endothelial NO synthase |
| ER/SR | endoplasmic/sarcoplasmic reticulum |
| ETAR | endothelin A receptor |
| FGF | fibroblast growth factor |
| FoxO3 | forkhead box O-3 |
| GECI | genetically encoded Ca^2+^ indicator |
| GFAP | glial fibrillary acidic protein |
| GPCR | G-protein coupled receptor |
| GVU | the gliovascular unit |
| HD | Huntingtin’s disease |
| 20-HETE | 20-hydroxyeicosatetraenoic acid |
| HIF-1α | hypoxia-inducible factor-1alpha |
| H_2_S | hydrogen sulfide |
| 5-HT | 5-hydroxytryptamine |
| ICH | intracerebral hemorrhage |
| IGF-1 | insulin-like growth factor 1 |
| IGF-1R | insulin-like growth factor 1 receptor |
| IP3 | inositol 1,4,5-triphosphate |
| IP3R | inositol 1,4,5-trisphosphate receptors |
| ISF | Inter-stitial fluid |
| K_ATP_ | adenosine triphosphate-sensitive K^+^ channel |
| KIR | inwardly-rectifying K^+^ channel |
| LCBF | local cerebral blood flow |
| LPS | lipopolysaccharide |
| LVO | large vessel occlusion |
| MCI | mild cognitive impairment |
| mGluR | metabotropic glutamate receptor |
| MND | motor neuron disease |
| mPTP | mitochondrial permeability transition pore |
| MRA | magnetic resonance angiography |
| MRI | magnetic resonance imaging |
| mTOR | the mammalian target of rapamycin |
| NCCT | non contrast computed tomography |
| NCX | Na^+^/Ca^2+^ exchanger |
| NDDs | neurodevelopmental and neurodegenerative diseases |
| NFAT | nuclear factor of activated T-cells |
| NF-κB | nuclear factor kappa B |
| NG2 | neuron-glia antigen 2 |
| NMDA | n-methyl-d-aspartate |
| NMDAR | n-methyl-d-aspartate receptor |
| NO | nitric oxide |
| NOS | nitric oxide synthase |
| NPC | neural progenitor cells |
| NSC | neural stem cells |
| NRF-1 | nuclear respiratory factor 1 |
| NVU | the neurovascular unit |
| OGD | oxygen glucose deprivation |
| PD | Parkinson's disease |
| PDC | pyruvate dehydrogenase complex |
| PDGF-B | platelet-derived growth factor subunit B |
| PDGFR-β | platelet-derived growth factor receptor-beta |
| PGT | prostaglandin transporter |
| α1/2-AR | G-protein coupled alpha1/2-adrenergic receptor |
| PGC-1α | peroxisome proliferator-activated receptor-gamma coactivator 1alpha |
| PGES | prostaglandin E synthase |
| PINK1 | phosphatas-e and tensin-homolog-induced kinase 1 |
| PLA2 | phospholipase A2 |
| PLC | phospholipase C |
| PLD2 | phospholipase D2 |
| polyP | inorganic polyphosphate |
| PPI | protein-protein interaction |
| RAC | reactive astrocytes |
| RBC | red blood cells |
| ROS | reactive oxygen species |
| RTK | receptor tyrosine kinase |
| rtPA | recombinant tissue plasminogen activator |
| RyR | ryanodine receptor |
| SBP | systolic blood pressure |
| sGC | soluble guanylate cyclase |
| SK_Ca_ | small (intermediate)-conductance Ca^2+^-activated K^+^ channel |
| α-SMA | alpha smooth muscle actin |
| Smac/DIABLO | second mitochondria-derived activator of caspase/direct inhibitor of apoptosis-binding protein with low pI |
| S1PR | sphingosine-1-phosphate receptor |
| ROS | reactive oxygen species |
| TCCD | transcranial color-coded duplex |
| TCD | transcranial Doppler |
| TRPC3 | transient receptor potential cation channel subfamily C member 3 |
| TRPV4 | transient receptor potential cation channel subfamily V member 4 |
| VDAC | voltage-dependent anion channel |
| VEGF | vascular endothelial growth factor |
| VOCC | voltage-dependent (voltage-gated) Ca^2+^ channel |
| VSMC | vascular smooth muscle cells |
| WML | white matter lesions |
